# Squaramide—Naphthalimide Conjugates as “Turn-On” Fluorescent Sensors for Bromide Through an Aggregation-Disaggregation Approach

**DOI:** 10.3389/fchem.2019.00354

**Published:** 2019-05-22

**Authors:** Lokesh K. Kumawat, Anthony A. Abogunrin, Michelle Kickham, Jyotsna Pardeshi, Orla Fenelon, Martina Schroeder, Robert B. P. Elmes

**Affiliations:** ^1^Department of Chemistry, Maynooth University, National University of Ireland, Maynooth, Ireland; ^2^Department of Biology, Maynooth University, National University of Ireland, Maynooth, Ireland; ^3^Maynooth University Human Health Research Institute, Maynooth University, National University of Ireland, Maynooth, Ireland

**Keywords:** supramolecular chemistry, squaramide, 1, 8-naphthalimide, fluorescent sensor, anion recognition

## Abstract

The syntheses of two new squaramide-naphthalimide conjugates (**SQ1** and **SQ2**) are reported where both compounds have been shown to act as selective fluorescence “turn on” probes for bromide in aqueous DMSO solution through a disaggregation induced response. **SQ1** and **SQ2** displayed a large degree of self-aggregation in aqueous solution that is disrupted at increased temperature as studied by ^1^H NMR and Scanning Electron Microscopy (SEM). Moreover, the fluorescence behavior of both receptors was shown to be highly dependent upon the aggregation state and increasing temperature gave rise to a significant increase in fluorescence intensity. Moreover, this disaggregation induced emission (DIE) response was exploited for the selective recognition of certain halides, where the receptors gave rise to distinct responses related to the interaction of the various halide anions with the receptors. Addition of F^−^ rendered both compounds non-emissive; thought to be due to a deprotonation event while, surprisingly, Br^−^ resulted in a dramatic 500–600% fluorescence enhancement thought to be due to a disruption of compound aggregation and allowing the monomeric receptors to dominate in solution. Furthermore, optical sensing parameters such as limits of detection and binding constant of probes were also measured toward the various halides (F^−^, Cl^−^, Br^−^, and I^−^) where both **SQ1** and **SQ2** were found to sense halides with adequate sensitivity to measure μM levels of halide contamination. Finally, initial studies in a human cell line were also conducted where it was observed that both compounds are capable of being taken up by HeLa cells, exhibiting intracellular fluorescence as measured by both confocal microscopy and flow cytometry. Finally, using flow cytometry we were also able to show that cells treated with NaBr exhibited a demonstrable spectroscopic response when treated with either **SQ1** or **SQ2**.

**Graphical Abstract:**
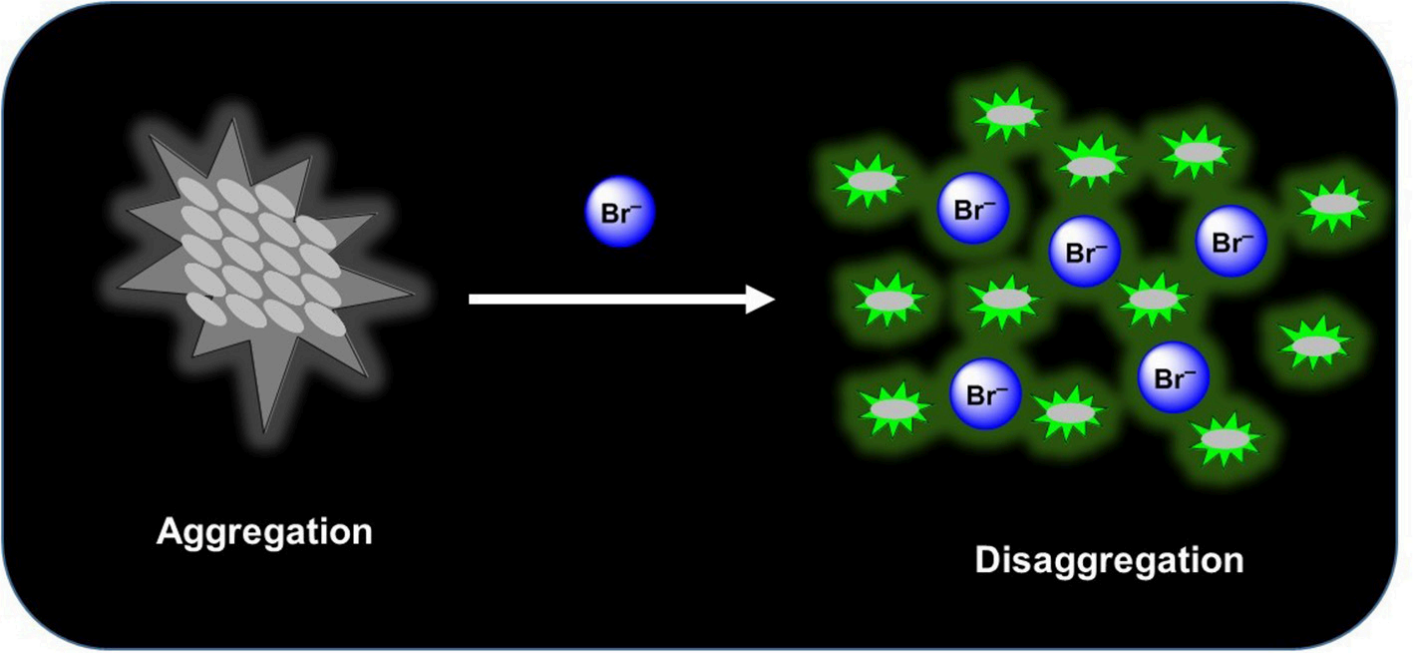

## Introduction

Anion sensing has been a key area of supramolecular chemistry since the introduction of the field in the early 1970's (Beer and Gale, 2001; Gunnlaugsson et al., 2006; Duke et al., 2010; Busschaert et al., 2015; Gale et al., 2016; Langton et al., 2016). In particular the recognition and sensing of halides has garnered considerable research interest due to their prevalence in biological and ecological settings (Verkman, 1990; Cametti and Rissanen, 2009, 2013; Evans and Beer, 2014; Ashton et al., 2015). While chloride and fluoride have captivated much of the research interest due to their major roles in cellular homeostasis, acid/base equilibria, and pollution concerns other halides such as bromide have been largely ignored. This is surprising given the clinical symptoms of bromide intoxication (also known as bromism), a once common disease where large doses of bromide were found to impair neuronal transmission and cause nausea and vomiting, abdominal pain, coma, and paralysis (Olson and System, 2004). Although now rare, bromism cases continue to be reported due to non-prescription bromide-based medications being available over the counter where a recent case resulted in extremely high levels of bromide intoxication (serum bromide level of 1,717 mg/L) (Hoizey et al., 2003). Moreover, from an environmental standpoint, bromide pollution can result in reaction between chlorine and naturally occurring organic matter in drinking-water, forming brominated, and mixed chloro-bromo by products such as trihalomethanes or halogenated acetic acids (Gribble, 2004). Indeed, the World Health Organization reports that while low levels of bromide pollution do not seem to have detrimental effects on humans or animals (World Health Organization, 2009), bromate (formed in water during ozonation) is a known carcinogen (World Health Organization, 2005). However, the selective binding and sensing of bromide is made challenging by its intermediate size and hydration energy between chloride and iodide (Marcus, 1991), and reported examples in the literature remain scarce (Kang and Kim, 2005; Suksai et al., 2005; Vlascici et al., 2018). One of the few examples, reported by Qian and co-workers, developed a rhodamine based fluorescent probe capable of selective sensing of iodide and bromide in aqueous solution based on a metal ion removal and anion ligand exchange mechanism (Xu et al., 2012). Nyokong and co-workers reported the use of GSH-capped quantum dots (QDs) covalently linked to a nickel tetraamino-phthalocyanine complex where the covalent binding of the QDs to the Ni complex induced fluorescence quenching before with introduction of Br– restoring the fluorescence (Adegoke and Nyokong, 2013). Most recently, Beer and co-workers developed a redox-active ferrocene functionalized rotaxane with a halogen bonding anion binding site that was capable of selective Br^−^ sensing over Cl^−^ in the presence of water as measured by ^1^H NMR and electrochemical measurements (Lim and Beer, 2019).

Much of the literature surrounding anion sensors relies on a binding event that disrupts/enhances some type of electron transfer mechanism such as photoinduced electron transfer (PET), internal charge transfer (ICT), excited state intramolecular proton transfer (ESIPT), fluorescence resonance energy transfer (FRET), etc. to yield a measurable fluorescence response (de Silva et al., 1997; Wu et al., 2011; Fan et al., 2013; Lee et al., 2015; Kumawat et al., 2017; Sedgwick et al., 2018). Some more recent reports take advantage of aggregation induced emission (AIE) (Hong et al., 2011), whereby recognition of an anion causes aggregation of the sensor thereby reducing molecular rotation and inducing large fluorescence perturbations (Peng et al., 2009; Ma et al., 2019). The opposite sensing mechanism, disaggregation induced emission (DIE), where dis-assembly of a non-fluorescent aggregate releases individual fluorescent molecules has received much less attention even though the process from aggregation to disaggregation generally causes a recovery or enhancement of fluorescence signals, and thus provides a useful method to design “turn-on” probes (Zhai et al., 2014). Recognition events that induce a fluorescence increase are preferable from the perspective of industrial/medical application where such a facile “turn-on” response can give naked-eye real-time information. Indeed, a recent report from O'Shea and co-workers detailed the use of an amphiphilic BF_2_-azadipyrromethene (NIR-AZA) DIE probe where a membrane selective fluorescence “off to on” switching event allowed visualization of dynamic cellular events in real-time (Wu et al., 2018). Here the authors exemplify how DIE can be exploited to produce a signal in response to a biological event specific to the plasma membrane, allowing real-time visualization of cell-cell contacts through pairs of filopodia.

**Figure 1:**
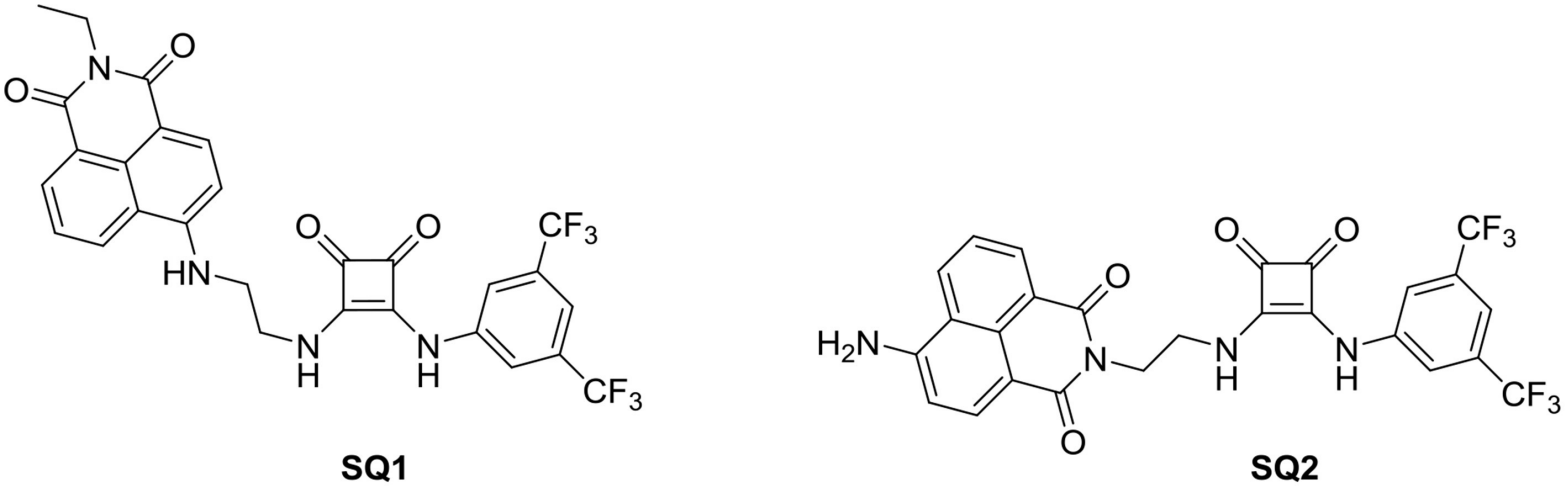
Chemical structures of probes **SQ1** and **SQ2**.

**Scheme 1:**
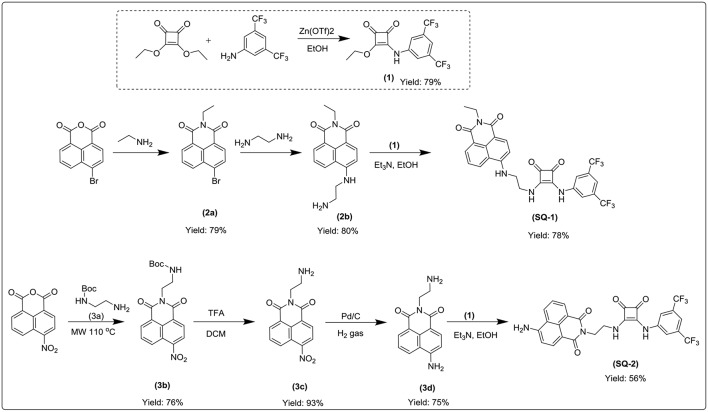
Syntheses of **SQ1** and **SQ2**.

From our work on squaramides we are familiar with the self-assembly/aggregation of these molecules due to bidirectional H-bonding interactions in addition to π-π stacking brought about by the aromatic cyclobutenedione ring (Elmes et al., 2013, 2014, 2015; Busschaert et al., 2014; Elmes and Jolliffe, 2014; Qin et al., 2016; Marchetti et al., 2018). Moreover, we have also gained considerable experience working with the naphthalimide fluorophore which has fluorescence properties that are highly dependent upon aggregation (Elmes and Gunnlaugsson, 2010; Elmes et al., 2012; Ryan et al., 2012, 2015; Ao et al., 2017). Indeed, Scanlan, Gunnlaugsson and co-workers have recently shown glycosylated naphthalimide and naphthalimide Tröger's bases that act as fluorescent aggregation probes for the Con A protein where both structures self-assemble in solution to form supramolecular structures by head-to-tail π-π stacking and extended hydrogen bonding interactions (Calatrava-Pérez et al., 2019). Inspired by this work, we envisaged that a naphthalide-squaramide conjugate may also aggregate efficiently in solution and such aggregation behavior may be modulated upon anion recognition where a disruption of a H-bonding network would reverse self-assembly. Thus, we set about designing a small family of squaramide-naphthalimide conjugates in which we wished to vary the position of the squaramide in relation to the naphthalimide to place the squaramide at either the “head” or the “tail” of the structure (Figure 1). As shown in Scheme 1 our design incorporated a short linker with a significantly hydrophobic side arm that we expected may aid in the aggregation behavior of the sensors in polar solvents.

Herein, we report the synthesis of compounds **SQ1** and **SQ2** where we have exploited their aggregation behavior and found differing fluorescence responses to various halides in solution. We have discovered that both **SQ1** and **SQ2** behave as DIE based sensors in solution show differing responses to various anions but with an unexpectedly selective fluorescence “turn on” response to bromide. Moreover, initial results obtained with the HeLa cell line demonstrate that the effect can also be observed in the complex biological environment of a human cell.

## Results and Discussions

### Synthesis

The syntheses of compounds **SQ1** and **SQ2** were achieved using the synthetic pathway outlined in Scheme 1. Briefly, **SQ1** was synthesized by amination of 4-bromo-1,8-naphthalimide **2a** using ethylenediamine before nucleophilic addition of the resulting intermediate **2b** to 3,5-bis(trifluoromethyl)phenyl squarate monoester **1** to yield **SQ1** in 78% yield. Similarly, **SQ2** was formed from an initial reaction of 4-nitro-1,8-naphthalic anhydride **3** with *N*-Boc-ethylenediamine **3a** to yield intermediate **3b** before subsequent TFA mediated deprotection of the Boc group **3c** and catalytic reduction of the nitro group yielded compound **3d** that was also reacted with 3,5-bis(trifluoromethyl)phenyl squarate monoester **1** to yield **SQ2** in 56% yield. All compounds and intermediates were fully characterized by ^1^H NMR, ^13^C NMR, HRMS and IR spectroscopy (see Supporting Information). However, during NMR characterization it was noted that the ^1^H NMR spectra of both **SQ1** and **SQ2** gave rise to complex spectra exhibiting significantly broadened peaks. Indeed, as discussed above, it is well-known that squaramides benefit from several characteristics that make them amenable for use in self-assembled materials, in particular their structural rigidity, aromaticity and ability to form strong two-dimensional hydrogen bonds (Storer et al., 2011; Wurm and Klok, 2013). Similarly, naphthalimides, with their extended planar, aromatic structure are well known to partake in π-π stacking; a characteristic that renders the photophysical properties of napthalimides sensitive to such stacking events (Duke et al., 2010; Banerjee et al., 2013). In order to investigate the self-assembly behavior of **SQ1** and **SQ2** several techniques such as ^1^H NMR, fluorescence measurements and SEM were utilized.

### Aggregation Behavior Measured by ^1^H NMR and SEM Spectroscopy

**Figure 2:**
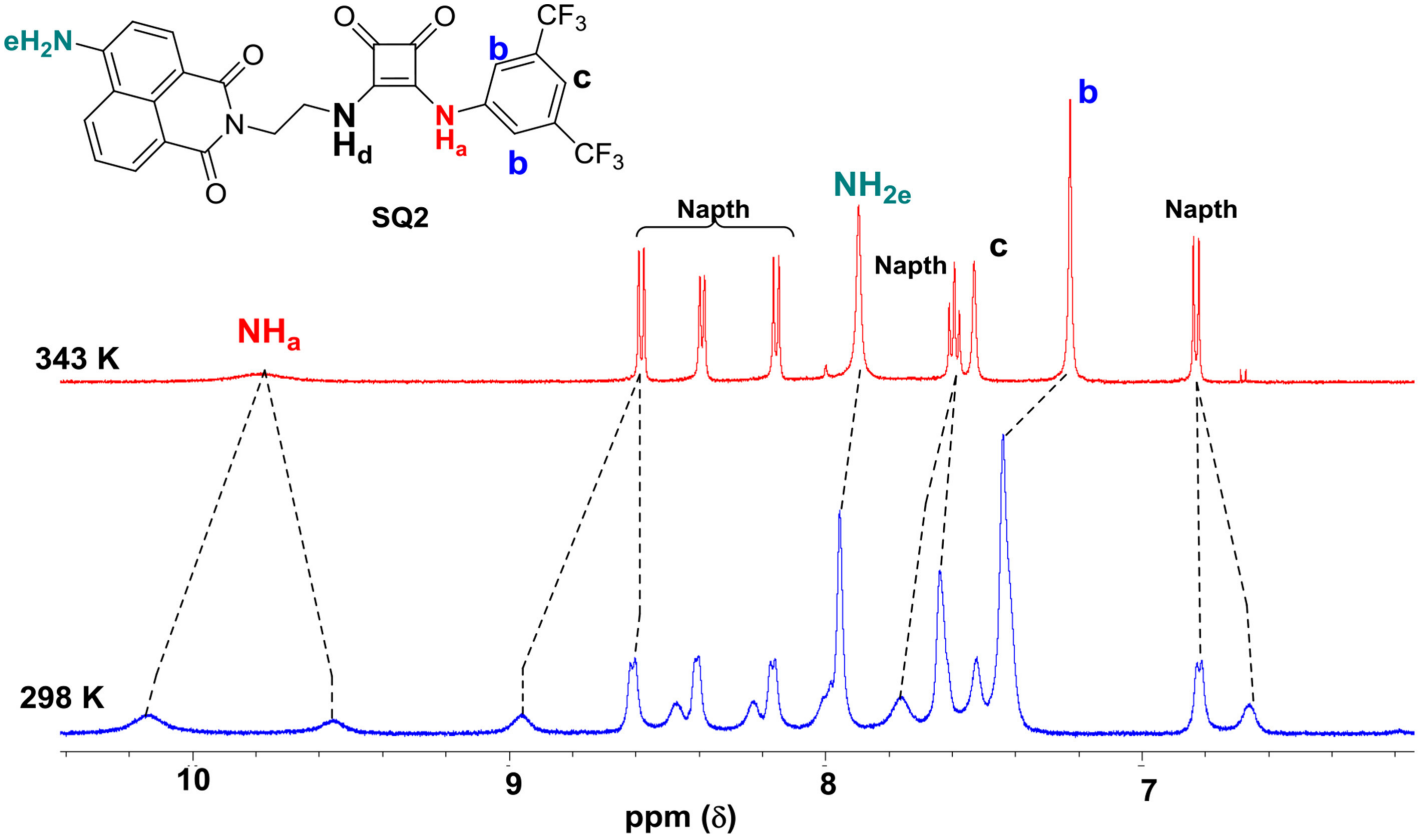
Disaggregation response of probes **SQ2** (5.0 × 10^−3^ M) at different temperatures. in DMSO-d_6_.

The ^1^H NMR spectra of **SQ1** and **SQ2** in *d6*-DMSO was measured at both 298 and 343 K. Both spectra clearly showed substantial differences between room and high temperatures where the spectra of **SQ1** and **SQ2** at 298 K appear broad and complex, as discussed above, signifying some degree of aggregation is occurring. Conversely, upon heating the samples to 343 K large changes in the spectra are observed whereby the signals become sharp and well-resolved allowing complete characterization of all ^1^H signals at this temperature. For example, Figure 2 exemplifies the apparent differences between the ^1^H NMR spectrum of **SQ2** at 298 and 343 K. Interestingly, signals associated with the naphthalimide moiety appear to resolve from two broad signals per proton to become one well-resolved signal for each proton. Moreover, the signals associated with the 3,5-bis(trifluoromethyl)phenyl portion of the molecule appear to undergo significant chemical shift changes, particularly the protons at the 2 and 6 position (H_b_) (Δδ = 105 Hz. Similarly, the CH_2_ directly attached to the squaramide moiety also resolves from two signals to one but does not fully resolve to the expected multiplicity and remains broad (Figure S20). Perhaps most striking is the fact that the squaramide NH signals appear as two distinct signals in the ^1^H NMR (10.1 and 9.56 ppm) at 298 K but are resolved to one broad signal at 9.78 ppm at 343 K. Taken together the evidence from the above study suggests that the entire molecule is taking part in aggregation behavior with the squaramide, naphthalimide and 3,5-bis(trifluoromethyl)phenyl portions all playing a role. Similar behavior was observed in the cases of both **SQ1** and **SQ2** (Figure S19).

**Figure 3:**
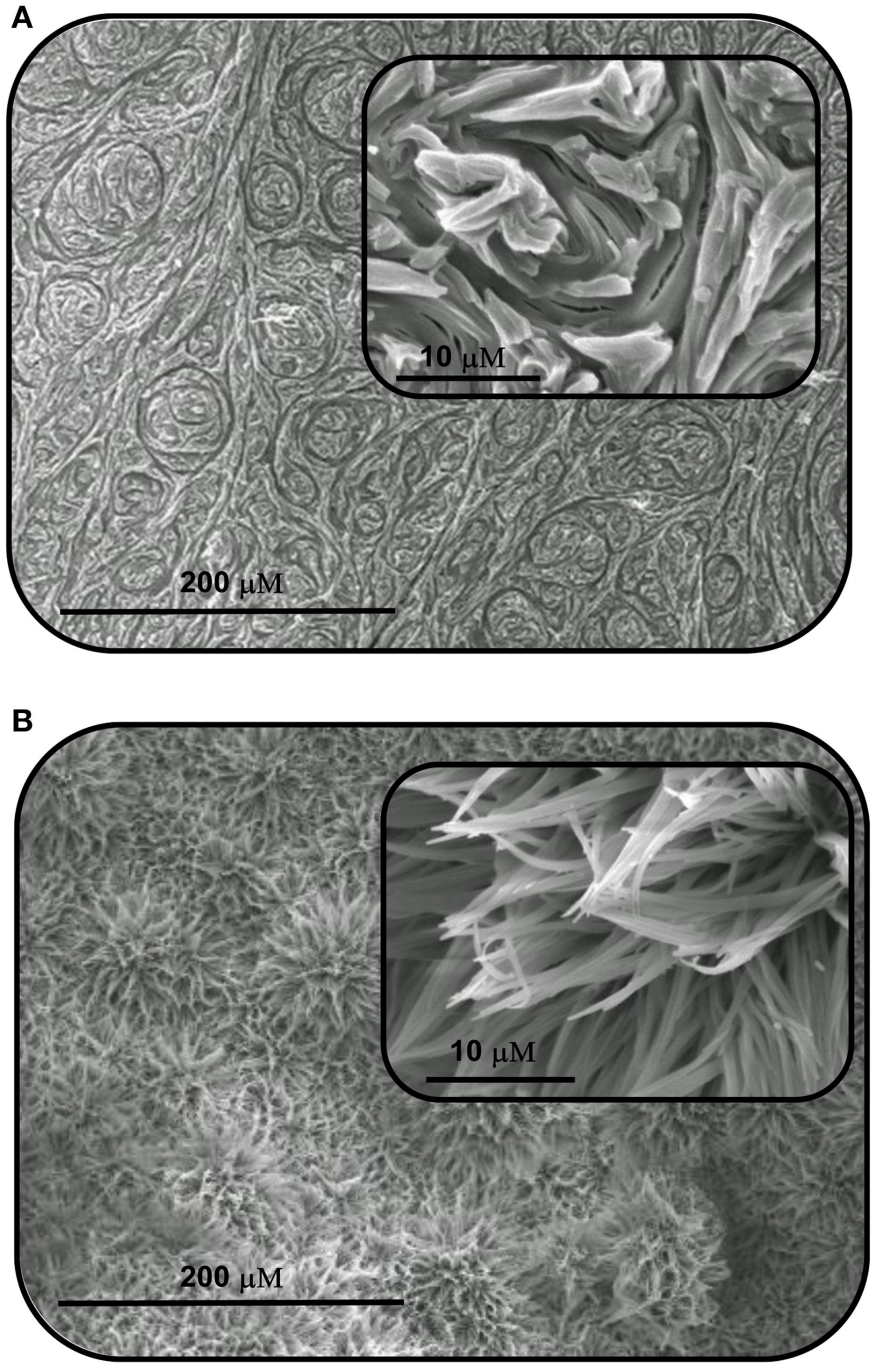
Scanning electron microscopy (SEM) images of the aggregates formed by self-assembly of **(A) SQ1** and **(B) SQ2** in DMSO.

In order to further probe the aggregation characteristics of both compound, attempts were made to grow crystals from concentrated DMSO solutions. Unfortunately, in our hands the compounds did not crystallize and instead formed what appeared to be amorphous solids. The morphological features of these solids were thus analyzed by scanning electron microscopy (SEM), and as shown in Figure 3, the SEM images exhibit interesting and distinct morphology on the nanoscale. **SQ1** appeared as “swirls” that propagate throughout the entire material. Conversely, **SQ2** formed showed a sponge like structure that at higher magnification appears to be composed of small nanofilaments. Although firm conclusions cannot be drawn on the exact molecular interactions that give rise to such interesting morphology it is clear that a high degree of aggregation is occurring that appears to be in an ordered fashion to give rise to such patterns.

**Figure 4:**
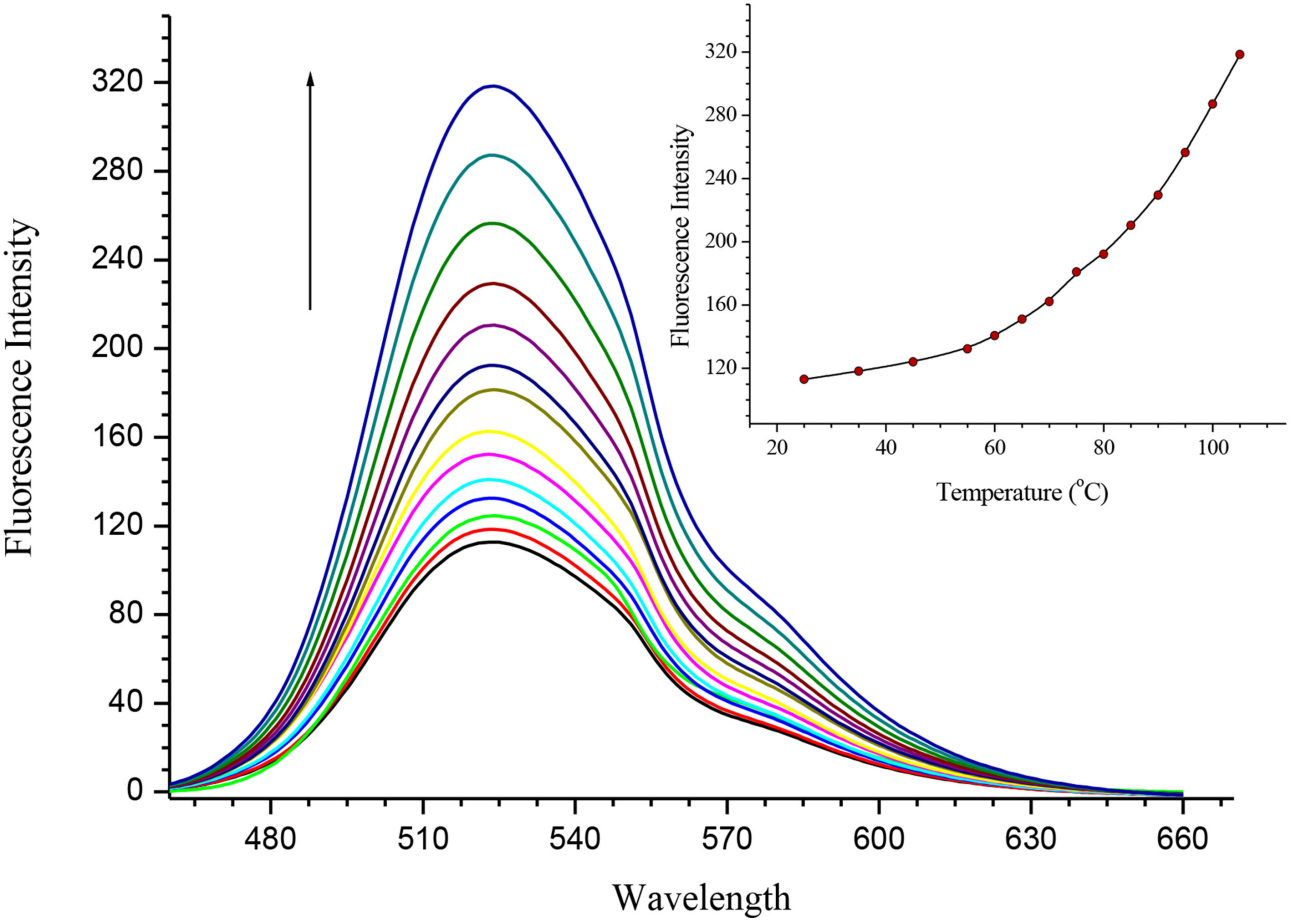
Fluorescence temperature study demonstrating the disaggregation response of **SQ2** (5.0 × 10^−6^ M) in 5% aq. DMSO from 25° to 110°C.

Both **SQ1** and **SQ2** were examined using UV/Vis spectroscopy and fluorescence emission spectroscopy. The UV/Vis absorption spectrum of **SQ1** in DMSO showed three absorption maxima at 280, 340, and 445 nm with extinction coefficient values of 32,642, 19,559, and 15,439 M^−1^ respectively. Similarly, **SQ2** showed an almost identical spectrum to **SQ1** with maxima also at 280, 340, and 445 nm and with extinction coefficients of 34,785, 20,405, and 13,717 M^−1^, respectively. Furthermore, both compounds exhibited fluorescence emission at *ca*. 525 nm. With the aggregation behavior observed in the previous section, we also undertook a thermal study to investigate if the fluorescence of **SQ1** and **SQ2** could be modulated by disaggregation. A temperature dependent fluorescence study was thus undertaken in 5% aq. DMSO. As seen in Figure 4, both **SQ1** and **SQ2** exhibited a sharp increase in fluorescence 220–300% as a function of temperature. These results support the ^1^H NMR results above where we suggest that both **SQ1** and **SQ2** aggregate in solution but, upon heating, disassemble thus allowing the fluorescence intensity to increase. Interestingly, upon cooling the emission does not decrease to its original value. This may suggest that once disrupted the self-aggregation behavior is not reversible under these conditions (Figure S23). A fluorescence dilution study was also conducted, where it was observed that the fluorescence intensity of both **SQ1** and **SQ2** at 525 nm is linear to concentration (from 0.05 to 5 μM) and suggests that aggregation occurs at very low concentrations (Figure S24).

With the observed properties exhibited by **SQ1** and **SQ2** and the known propensity of squaramides to bind strongly to halides and other anionic species (Prohens et al., 1998, 2001; Piña et al., 2008; Amendola et al., 2010; Busschaert et al., 2012; Delgado-Pinar et al., 2012; Jin et al., 2013), we expected that introduction of anionic analytes may also disrupt the self-aggregation of the **SQ1** and **SQ2**. If indeed disruption was to occur, we expected some modulation of their photophysical properties would result and potentially give rise to a new class of anion sensors based on disaggregation.

### Anion Induced Disaggregation

**Figure 5:**
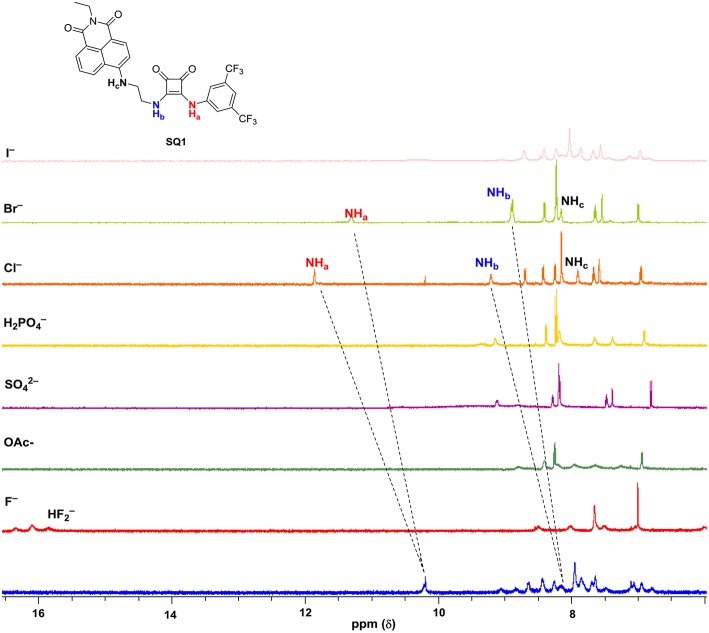
^1^H NMR stackplot of **SQ1** with various anions (30 equivalents) in 0.5% H_2_O in DMSO-d_6_ at 298 K.

To investigate the ability of anions to disrupt the self-aggregation of **SQ1** and **SQ2** a series of ^1^H NMR experiments were carried out. Initial qualitative measurements were undertaken using a screening experiment in which 30 equiv. of several anions (AcO^−^, H_2_${\text{PO}}_{4}^{-}$, ${\text{SO}}_{4}^{2-}$, F^−^, Cl^−^, Br^−^, and I^−^ as their tetrabutylammonium salts) were added to the receptors in solution (0.5% H_2_O in DMSO-d_6_). These preliminary results showed significant changes of the spectra of both **SQ1** and **SQ2**. Dramatic changes were observed in the ^1^H NMR spectra of both receptors in the presence of AcO^−^, F^−^, H_2_${\text{PO}}_{4}^{-}$, and ${\text{SO}}_{4}^{2-}$ where addition of these anions led to the disappearance of the NH signal (H_a_) as shown in Figure 5 for **SQ2** and also either broadened (AcO^−^ and F^−^) or sharpened (H_2_${\text{PO}}_{4}^{-}$ and ${\text{SO}}_{4}^{2-}$).

Stark color changes of the solutions of **SQ1** and **SQ2** were observed upon the addition of F^−^ from yellow to red. The disappearance of the NH signal (H_a_) and the stark color change suggests that deprotonation of the squaramide/naphthalimide may be responsible. We further confirmed this deprotonation behavior by observation of bifluoride (${\text{HF}}_{2}^{-}$) in the ^1^H NMR spectra of **SQ1** and **SQ2** upon the addition of F^−^ as shown in Figure 5 for receptor **SQ1**. Moreover, distinct changes were observed in the ^1^H NMR spectra of **SQ1** and **SQ2** upon the addition of Cl^−^ and Br^−^. For example, with **SQ2** these halides led to a large downfield shift of the NH signal (H_a_) from 10.2 to 12.1 ppm for Cl^−^ and to 11.1 ppm for Br^−^ coupled with a significantly sharpened signal. Similar behavior was observed for **SQ1** (see Figure S25). The large downfield shift of the NH signal suggests a classical H-bonding interaction between Cl^−^ and Br^−^ and the NH protons of the squaramides. To further investigate the binding interaction of halides with **SQ1** and **SQ2** more detailed ^1^H NMR spectroscopic titrations were carried out with halides F^−^, Cl^−^, Br^−^, and I^−^ as their tetrabutylammonium salts with the resulting data fit to a 1:1 binding model using the open access BindFit software program (Thordarson, 2011; Lowe et al., 2012; Brynn Hibbert and Thordarson, 2016) to provide the apparent stability constants (K_a_), which are summarized in Table 1.

**Table 1:** Summary of the halide association constants K_a_ (M^−1^) of receptors **SQ1** and **SQ2** in DMSO-d_6_ at 273 K according to the ^1^H NMR titration data.

**Receptor**	***K**_***a***_* **(M**^**−1**^**) (% Error)**			
	**F^**−**^**	**Cl^**−**^**	**Br^**−**^**	**I^**−**^**
**SQ1**	–^a^	489 (6%)	165 (10%)	–^b^
**SQ2**	–^a^	221 (9%)	99 (9%)	–^b^

a *Addition of F^−^ resulted in deprotonation and prevented an association constant from being determined*.

b *Spectral changes were too minor to provide an accurate association constant. A series of equilibria may occur in solution under the reported conditions (e.g., aggregate disruption, anion binding to the monomer; anion binding to the aggregate etc.) thus these data, that have been fitted to a 1:1 binding model, are included to give a comparison of analogous squaramide anion receptors previously reported Busschaert et al., 2012;Bao et al., 2018*.

**Figure 6:**
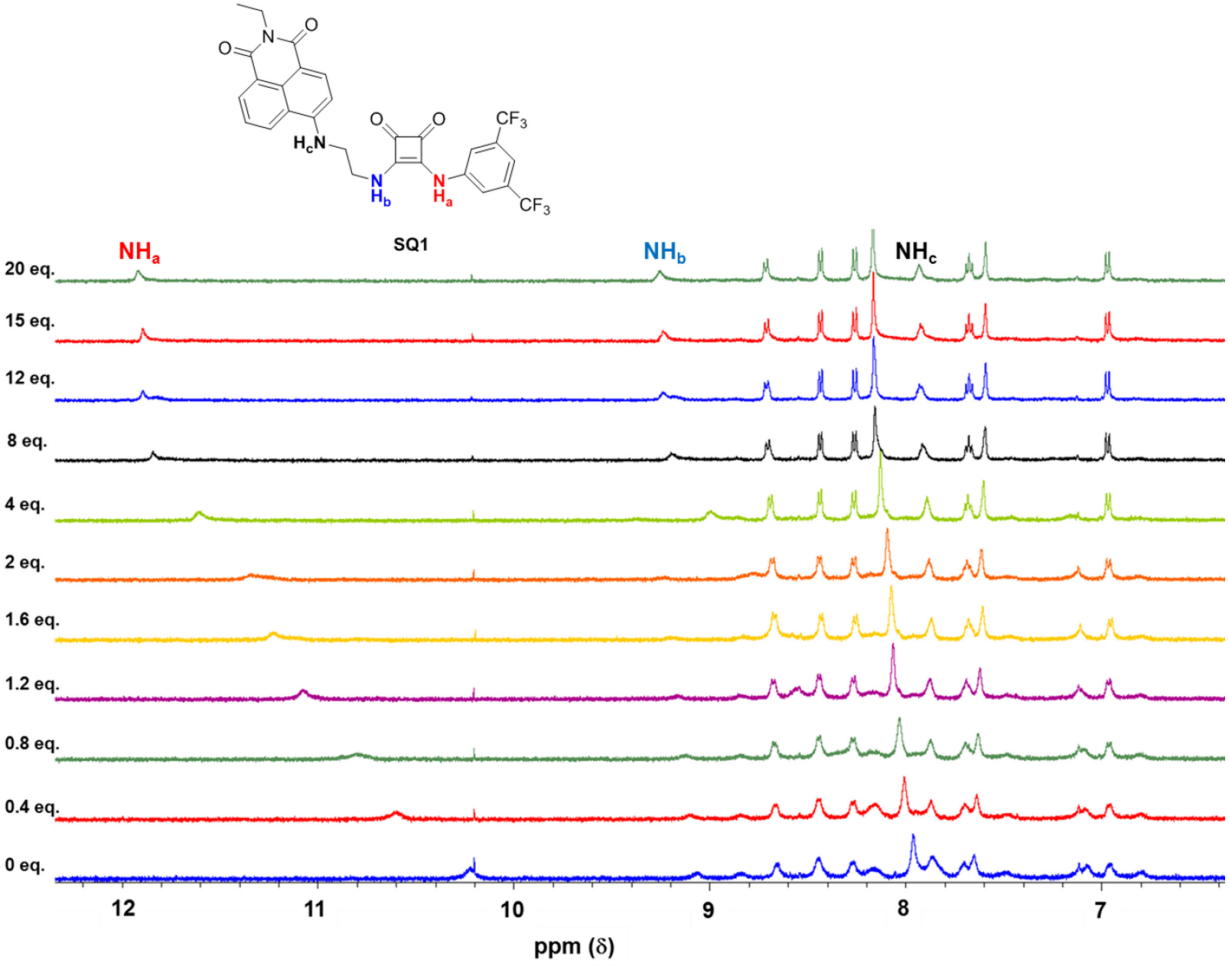
Changes in the ^1^H NMR spectrum (NH_a_, NH_b_, NH_c_ identified) of **SQ1** upon the addition 0–20 equivalents of anions as tetrabutylammonium (TBA) chloride in DMSO-d_6_.

As an example, Figure 6 shows the changes observed in the ^1^H NMR spectrum of **SQ1** upon the addition of Cl^−^ (20 equiv.). A gradual downfield shift of the NH signal (H_a_) from 10.2 to 11.8 ppm in the ^1^H NMR spectrum was observed upon the increasing concentration of Cl^−^. These changes together with increased spectral resolution observed over the entire spectrum upon the addition of Cl^−^, clearly support a classical H-bonding interaction between Cl and the NH protons (H_a_) of the receptors.

Overall, both **SQ1** and **SQ2** were found to bind to Cl^−^ and Br^−^ with moderate affinities with **SQ1** showing a slightly increased affinity for both anions compared to **SQ2** where the position of the squaramide at the “tail” of the naphthalimide seems to be optimal for both anions. Similarly, both receptors exhibited a preference for Cl^−^ over Br^−^ while I^−^ showed an almost complete lack of binding in both cases. Conversely, as previously discussed F^−^ resulted in receptor deprotonation thus the data could not be fit to a suitable ^1^H NMR binding model. Analysis of the ^1^H NMR spectra of the complexes also provided further evidence for the disaggregation of **SQ1** and **SQ2** in the presence of Cl^−^ and Br^−^ where the bound receptors gave rise to a well-resolved spectrum with sharp signals that could be clearly attributed to the proposed target structures. In addition, the signal for the CH_2_ protons directly attached to the squaramide moiety in both cases resolved from two broad signals to one single signal with significantly improved resolution (Figures S20, S21).

While conducting the ^1^H NMR experiments we also noticed samples containing Cl^−^ and Br^−^ exhibited significantly more intense fluorescence than the parent receptors in solution when irradiated with UV light. This chance observation led us to investigate the fluorescence behavior of **SQ1** and **SQ2** upon titration with the halides in more detail.

**Figure 7:**
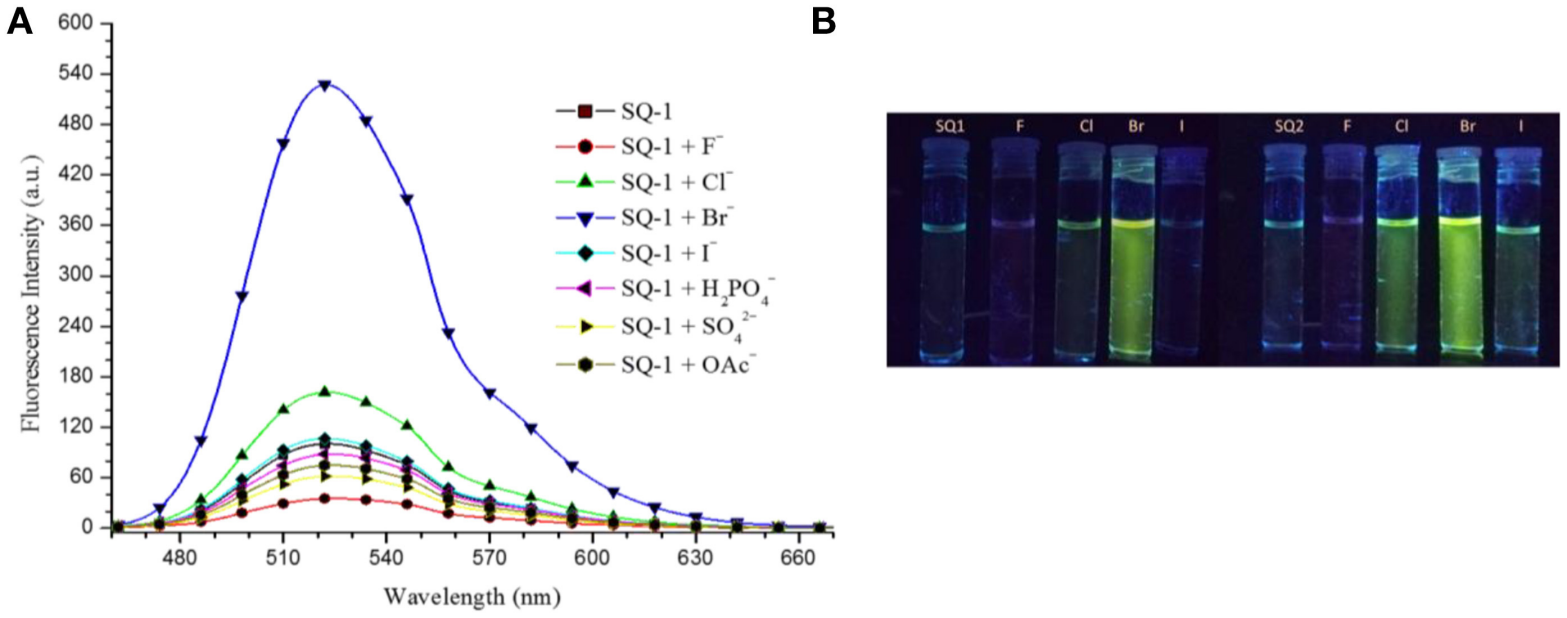
**(A)** Fluorescence response of **SQ1** (5 μM) in 5% aq. DMSO toward different TBA anions (20 mM). (λ_ex_. 435 nm). **(B)** Fluorescence response of **SQ1** and **SQ2** toward various halides in 5% aq DMSO after UV illumination as seen by the naked eye.

Having observed such stark changes in the ^1^H NMR titrations of **SQ1** and **SQ2** in the presence of halides we next wished to investigate their excited state properties in the presence of F^−^, Cl^−^, Br^−^, and I^−^. Titrations were performed in 5% aq. DMSO with addition of aliquots of the anions as their tetrabutylammonium salts. Both receptors exhibited emission with maxima at ca. 525 nm in solution. As shown in Figure 7 additions of the halides resulted in varying effects. In the presence of I^−^ minor changes were observed where a small increase in emission intensity (20–25%) was observed up to a concentration of 20 mM. Addition of F^−^ on the other hand, was found to result in a large decrease in emission intensity (80–85%) culminating in the both **SQ1** and **SQ2** largely appearing as non-fluorescent. We ascribe this behavior to the deprotonation observed in the NMR titrations and similar behavior was also observed in the presence of basic non-halide anions such as AcO^−^, H_2_${\text{PO}}_{4}^{-}$, ${\text{SO}}_{4}^{2-}$. Most strikingly, however, was the observation of large fluorescence increases in the presence of both Cl^−^ and Br^−^ where the addition of Cl^−^ resulted in a 98 and 170% enhancement of emission from **SQ1** and **SQ2**, respectively, while, unexpectedly, Br^−^ resulted in a 500 and 600% enhancement of emission from **SQ1** and **SQ2**, respectively. From the previous temperature dependent fluorescence study, we suggest that the emission enhancement is as a result of the disaggregation of **SQ1** and **SQ2** in solution where aggregation induced self-quenching is disrupted allowing the release of monomers in solution and thus a recovery in fluorescence. Moreover, these responses were also clearly visible to the naked eye under UV illumination (Figure 7B). In order to investigate the effect of water on the fluorescence response, qualitative titrations were also performed in non-aqueous DMSO and in 20% aq. DMSO. We observed that the selective response to Br^−^ in pure DMSO was similar to that seen in 5% aq DMSO while in 20% aq. DMSO the response was considerably less defined. These results suggest that water concentration has a significant effect on the aggregation behavior of both **SQ1** and **SQ2** and thus their ability to act as halide sensors in fully aqueous environments (Figures S27, 28).

**Figure 8:**
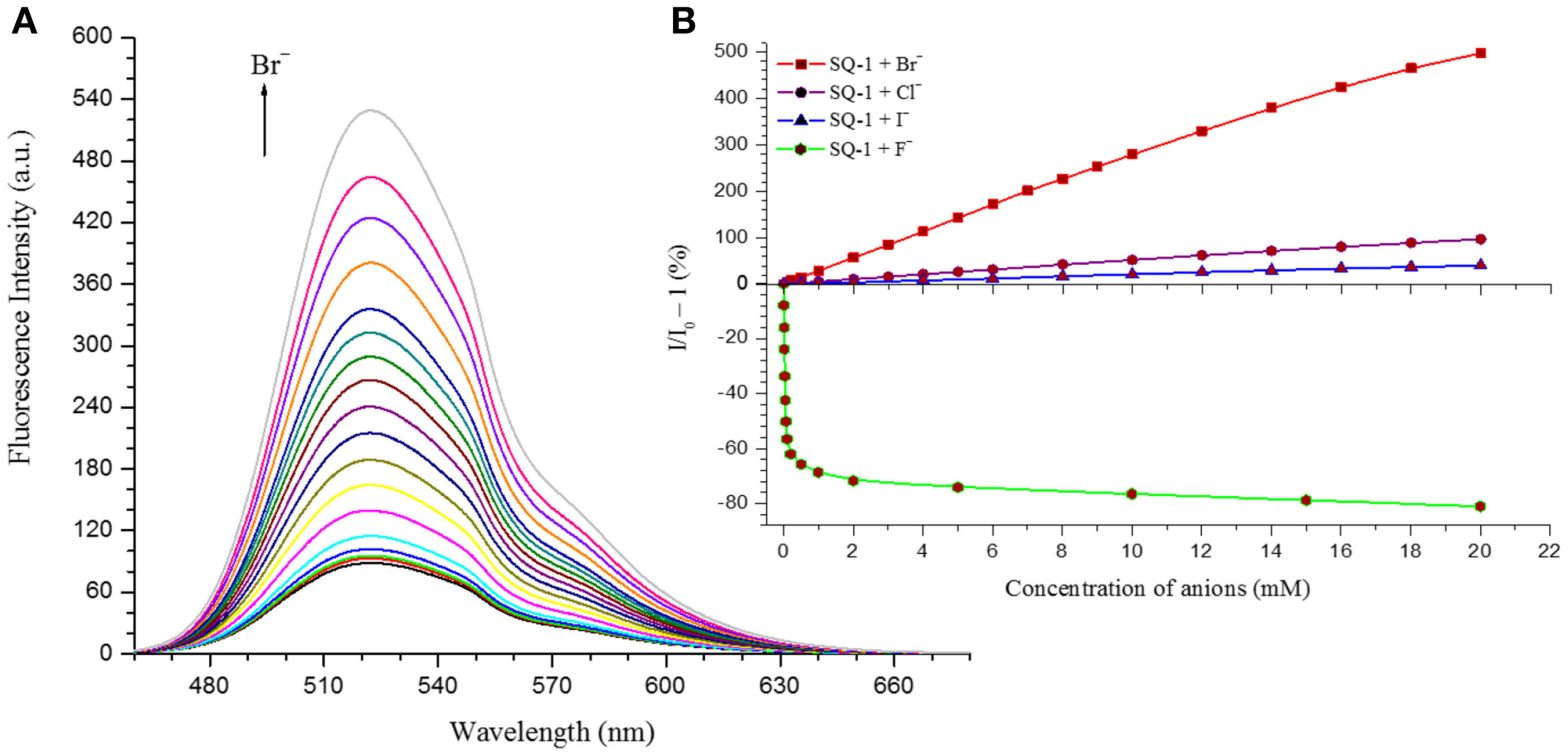
**(A)** Changes in the fluorescence response of **SQ1** in the presence of Br^−^ in 5% aq. DMSO **(B)** Relative fluorescence enhancement and quenching response of **SQ1** with different halides in 5% aq. DMSO.

Nevertheless, in order to investigate the sensing responses of **SQ1** and **SQ2** in more detail, fluorescence titrations were performed with 0.0–100.0 equivalent increments of halides in 5% aq. DMSO. As seen in the qualitative experiments the fluorescence intensity of both receptors is quenched by 80–85% upon addition of F^−^ while minor fluorescence enhancements are observed for I^−^. However, a larger degree of enhancement (98–170%) is observed upon addition of Cl^−^ and, most interestingly, we observed a 500–600% increase in fluorescence intensity centered at 525 nm upon addition of Br^−^ (Figure 8).

These results demonstrate that both **SQ1** and **SQ2** show a selective fluorescence response toward Br^−^ when compared against other halides. This result is particularly unexpected given the NMR studies above where the binding of **SQ1** and **SQ2** to Cl^−^ appears to be considerably stronger when measured by ^1^H NMR titration. Moreover, the desired “OFF-ON” fluorescence response paves the way for **SQ1** and **SQ2** to be used as selective bromide sensors with potential industrial/medical application. Limit of detection (LOD) values were estimated using a standard deviation method (LOD) = (3 S/m, in which S is the standard deviation of blank measurements, *n* = 11, and m is the slope of the linear equation) (Kumar et al., 2016), where **SQ1** and **SQ2** were found to exhibit LOD values in the μM range; values that are adequate to measure trace levels of halide contamination. A summary of the fluorescence quenching/enhancement characteristics and LOD values for **SQ1** and **SQ2** in the presence of the halides are listed in Table 2.

**Table 2:** Summary of the fluorescence quenching/enhancement characteristics and the Limit of Detection (LOD) values obtained for **SQ1** and **SQ2** in the presence of halides.

**Probe**	**Anions**	**Fluorescence response**	**Detection limit (LOD = 3σ/slope)**
**SQ1**	F^−^	80% Quenching	6.25 × 10^−6^ M
	Cl^−^	98% Enhancement	9.65 × 10^−5^ M
	Br^−^	500% Enhancement	1.33 × 10^−5^ M
	I^−^	20% enhancement	8.03 × 10^−4^ M
**SQ2**	F^−^	85% Quenching	5.58 × 10^−6^ M
	Cl^−^	170% Enhancement	7.86 × 10^−5^ M
	Br^−^	600% Enhancement	1.92 × 10^−5^ M
	I^−^	25% enhancement	5.51 × 10^−4^ M

### Evaluation of SQ1 and SQ2 in a Human Cell Line

**Figure 9:**
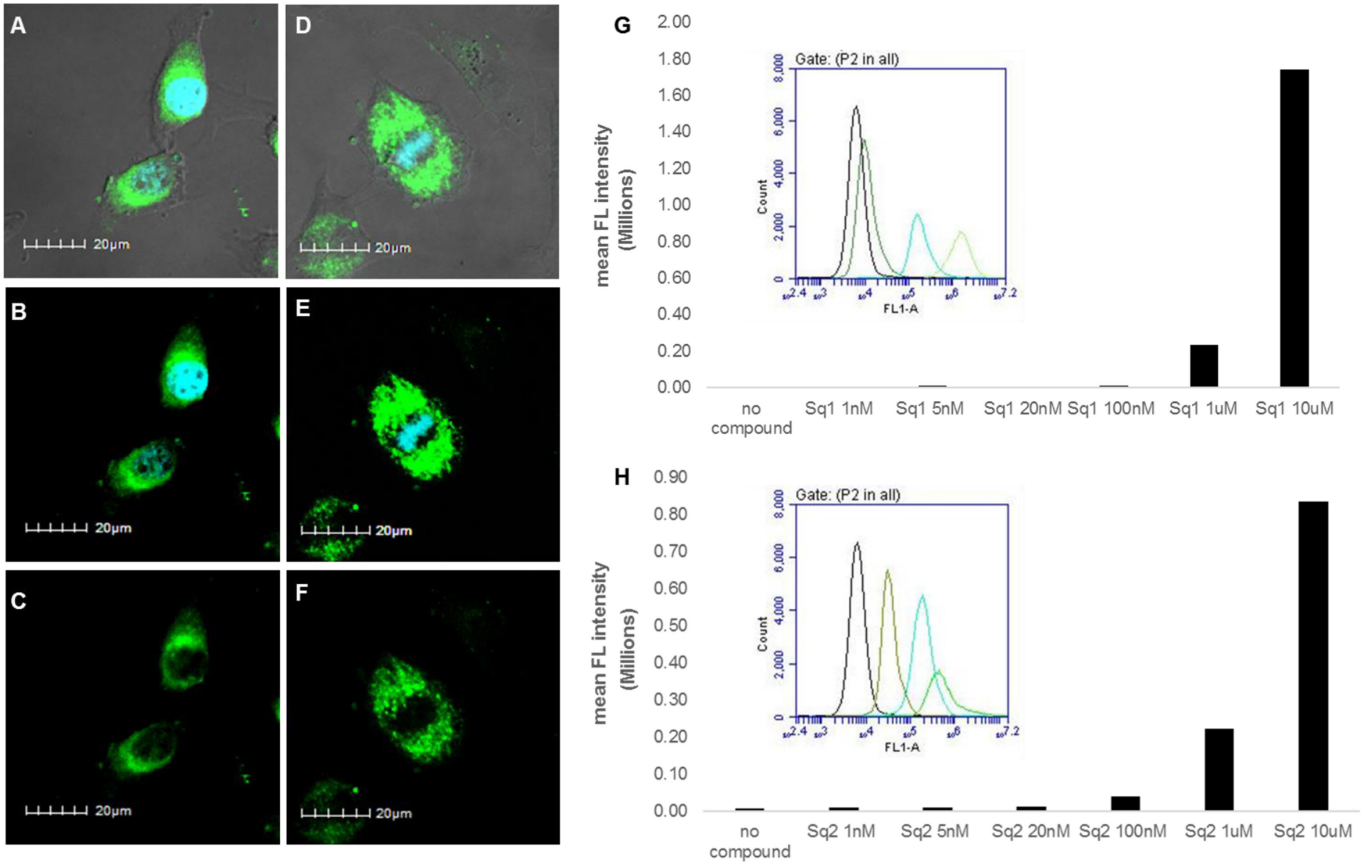
Uptake of **SQ1** and **SQ2** (5 μM) by HeLa cells. **(A–F)** shows images obtained by confocal microscopy. **(A)** shows an overlay of the bright field image of treated cells with the DAPI (4′,6-diamidino-2-phenylindole) signal (blue) and **SQ1** (green), **(B)** shows overlay of **SQ1** (green) and nuclear co-stain DAPI (blue), **(C)** shows **SQ1** fluorescence alone (green), **(D)** shows an overlay of the bright field image of treated cells with the DAPI signal (blue) and **SQ2** (green), **(B)** shows overlay of **SQ2** (green) and nuclear co-stain DAPI (blue), **(C)** shows **SQ2** fluorescence alone (green). Scale bars: 20 μm. Additional images displayed in the Supporting Information. **(G,H)** shows flow cytometry data for **SQ1** and **SQ2**, respectively. Bar charts show the mean fluorescence intensity (FL-1) obtained in a representative experiment (out of two) with cells treated with **SQ1** or **SQ2** over a wide range of concentrations (1 nm−10 μM). In addition, histogram plots for FL-1 are also shown to illustrate the clear shift in FL-1 for the entire cell population (untreated cells (black), 100 nM, 1, 10 uM).

With the observed spectroscopic responses and the potential application of **SQ1** and **SQ2** as bromide sensors we also wished to evaluate their biocompatibility in a human cell line. Gale and co-workers have recently reported analogous naphthalimide-squaramide conjugates for use as anion receptors and transmembrane anion transporters where they found that the most active anion transporter [which also contained a 3,5-bis(trifluoromethyl)phenyl squaramide moiety] was readily internalized in human lung carcinoma A549 cells and exhibited no toxicity up to a concentration of 100 μM as determined using a CCK-8 assay (Bao et al., 2018). Thus we set out to conduct an initial evaluation of **SQ1** and **SQ2** uptake and behavior in cells. Firstly, the ability of **SQ1** and **SQ2** to be internalized into cells was assessed using confocal microscopy in HeLa cells, a widely-used cervical carcinoma cell line. HeLa cells (0.5 × 10^5^) were incubated with either **SQ1** or **SQ2** (either 20, 5, or 1 μM) at 37°C for 1 h before the cells were fixed, and mounted for microscopy. Figure 9 shows examples of cells incubated with **SQ1** and **SQ2** at 5μM, demonstrating successful uptake, as all cells in the sample emitted strong green fluorescence. **SQ1** and **SQ2** appear to localize in the cytoplasm as co-localization studies with the nucleic acid stain DAPI (blue) suggest that neither **SQ1** nor **SQ2** enter the nucleus. Uptake of both compounds was also investigated in a more quantitative manner using flow cytometry where, again, both **SQ1** and **SQ2** were rapidly taken up by HeLa cells. Resulting histogram plots show an increase in mean fluorescence intensity in a concentration-dependent manner and a clear shift for the entire cell population, demonstrating that all cells successfully internalized the compounds (Figure 9).

**Figure 10:**
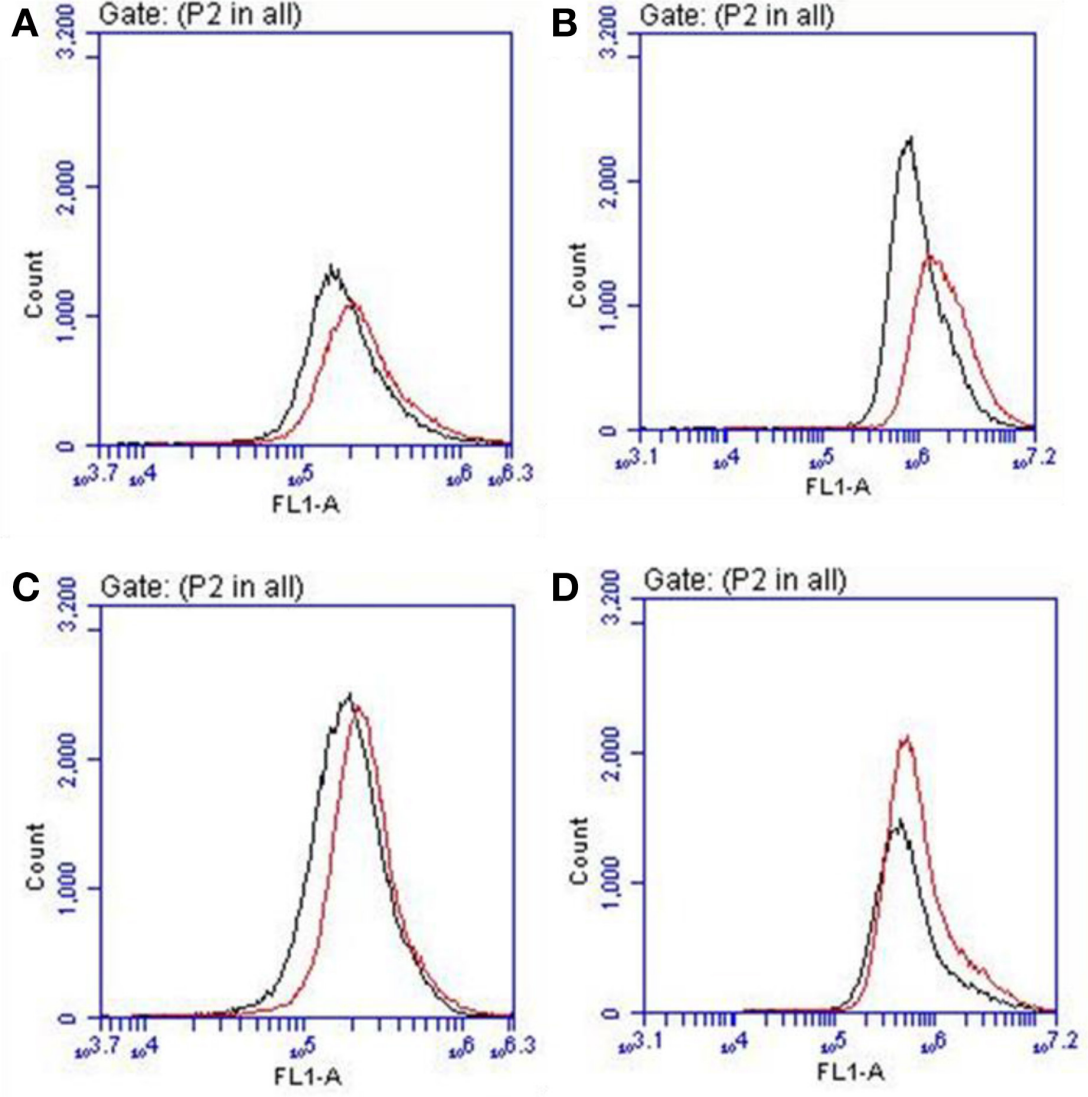
Flow cytometry histogram plots for HeLa cells incubated with **(A) SQ1** (1 μM), **(B) SQ1** (10 μM), **(C) SQ2** (1 μM), and **(D) SQ2** (10 μM) in the absence (black) or presence of NaBr (red).

Cellular viability in the presence of **SQ1** and **SQ2** was measured using an MTT assay where the results showed that the % viability of HeLa cells decreased in a dose-dependent manner in the presence of **SQ1** and **SQ2**, with **SQ2** showing higher levels of cytotoxicity compared to **SQ1**. **SQ1** appeared to be largely non-toxic up to 1 μM while **SQ2** at 1 μM showed % cell viability at 28% (±20%) (see Figure S42). Gale and co-workers reported no toxicity up to a concentration of 100 μM for their 1,8-naphthalimide–squaramide conjugates, while **SQ1**, which is structurally very similar to the compounds reported by Gale, was shown to be considerably more cytotoxic under these conditions (Bao et al., 2018). Finally, we wished to determine whether the Br^−^ sensing abilities of these compounds could be measured *in cellulo*. As mentioned above O'Shea (Wu et al., 2018) and others (Zhai et al., 2014) have already exploited a disaggregation approach to sensing in a biological environment, thus we sought to investigate if **SQ1** and **SQ2** could measure bromide contamination in cells. Indeed, as shown in Figure 10, flow cytometry analysis was able to show a further increase in fluorescence intensity for cells incubated with **SQ1** or **SQ2** in the presence of sodium bromide (50 mM) compared to those incubated with the squaramide receptors in the absence of sodium bromide.

## Conclusions

The field of molecular recognition continues to grow with ever more elegant receptors being reported that are capable of selective recognition for various anions using a diverse set of approaches. Indeed, with our experience of studying squaramide based probes we have attempted to uncover an alternative approach for selective detection of a somewhat neglected halide; Br^−^. In this study we have reported the synthesis of two new squaramide-naphthalimide conjugates based on a “head to head” or “head to tail” design. We initially discovered that both probes **SQ1** and **SQ2** displayed a large degree of self-aggregation in aqueous DMSO solution that could be disrupted at increased temperature as studied by ^1^H NMR and SEM. Moreover, we also discovered that the fluorescence behavior of both receptors was highly dependent upon the aggregation state and disruption of aggregation by increasing temperature gave rise to a significant increase in fluorescence intensity. We therefore exploited this disaggregation induced emission response for the recognition of halides, where we discovered that the receptors gave rise to distinct responses related to the interaction of the various halide anions with the receptors. Addition of F^−^ rendered both compounds non-emissive; thought to be due to a deprotonation event while, surprisingly, Br^−^ resulted in a dramatic 500–600% fluorescence enhancement thought to be due to a disruption of compound aggregation and allowing the monomeric receptors to dominate in solution. Furthermore, optical sensing parameters such as limits of detection and binding constant of probes were also measured toward the various halides (F^−^, Cl^−^, Br^−^, and I^−^) where both **SQ1** and **SQ2** were found to sense halides with adequate sensitivity to measure μM levels of halide contamination. Finally, initial studies in a human cell line were also conducted where it was observed that both compounds are easily taken up by HeLa cells, exhibiting strong intracellular fluorescence as measured by both confocal microscopy and flow cytometry. We were also able to demonstrate that **SQ1**- or **SQ2**- containing Hela cells treated with NaBr exhibited increased fluorescence intensity. While the observed increases in fluorescence intensity were modest in relation to those observed in the spectroscopic titrations described above, cell cytometry analysis allowed some distinction to be made. Given the complex environment of the cellular cytoplasm, the fully aqueous environment and the presence of intracellular Cl^−^ at high concentrations (ranging from 5 to 80 mM in mammalian cells) (Andersen, 2013) it is not surprising that the response is considerably less pronounced. Solvent effects are also likely to play a major role in the anion response. To the best of our knowledge, **SQ1** and **SQ2** are the first such fluorescent sensors capable of sensing Br^−^*in cellulo* and we are currently working toward sensors with an improved bromide sensitivity and cellular cytotoxicity profile.

## Experimental Section

### 3-((3,5-bis(trifluoromethyl)phenyl)amino)-4-ethoxycyclobut-3-ene-1,2-dione (1)

A solution of 3,5-bis(trifluoromethyl)aniline (1.835 mL, 11.75 mMol, 1eq) in EtOH (5 mL) was slowly added to a mixture of diethyl squarate (1.74 mL, 11.75 mMol, 1 eq) and zinc triflate (0.85 g, 2.35 mMol, 0.2 eq) in 5 mL EtOH. The reaction was stirred at room temperature overnight. The precipitate was collected by suction filtration and washed with EtOH and Et_2_O to yield the product as an off-white amorphous solid (3.28 g, 79%). ^**1**^**H NMR**, (DMSO-*d*_6_, 500.13 MHz) δ (ppm), J (Hz): 1.41 (t, *J* = 7.1, 3H, CH_3_), 4.79 (q, *J* = 7.1, 2H, CH_2_), 7.78 (s, 1H, phenylene H), 8.03 (s, 2H, phenylene H), 11.19 (br, 1H, NH); ^**13**^**C NMR** (DMSO-*d*_6_, 125.76 MHz): δ (ppm): 184.9, 179.7, 140.6, 131.7, 131.4, 131.2, 126.8, 124.6, 122.4, 119.9, 116.8, 70.5, 15.8; **HRMS** (ESI) calcd. for C_14_H_10_F_6_NO_3_ [M + H]^+^ 354.056, found 354.0554; **ν**_max_ **(KBr)/cm**^**−1**^**:** 3,254, 3,185, 3,100, 3,062, 3,004, 1,816, 1,717, 1,603, 1,476, 1,453, 1,278, 1,233, 1,170, 1,101, 1,042, 997, and 936.

### 6-bromo-2-ethyl-1H-benzo[de]isoquinoline-1,3(2H)-dione (2a)

4-Bromo-1,8-naphthalic anhydride (2.0 g, 7.33 mMol, 1 eq) was mixed with ethylamine (0.57 mL, 8.66 mMol, 1.2 eq) in EtOH (10 mL). The reaction mixture was refluxed at 80°C for 24 h. The precipitate was collected by suction filtration and washed with EtOH and Et_2_O to yield the product as a light tan amorphous solid (1.74 g, 79%). ^**1**^**H NMR**, (DMSO-*d*_6_, 500.13 MHz) δ (ppm), *J* (Hz): 1.21 (t, *J* = 7.0, 3H, CH_3_), 4.06 (q, *J* = 7.0, 2H, CH_2_), 7.99 (t, *J* = 8.0, 1H, phenylene H), 8.21 (d, *J* = 7.8, 1H, phenylene H), 8.32 (d, *J* = 7.8, 1H, phenylene H), 8.55 (q, *J* = 7.8, 2H, phenylene H); ^**13**^**C NMR** (DMSO-*d*_6_, 125.76 MHz): δ (ppm): 163.1, 163.1, 133.0, 132.0, 131.8, 131.4, 130.3, 129.5, 129.3, 128.8, 123.3, 122.5, 35.4, 13.5; **HRMS** (ESI) calcd. for C_28_H_20_Br_2_N_2_NaO_4_ [2M + Na]^+^ 628.968, found 628.9599; **ν**_max_ **(KBr)/cm**^**−1**^**:** 3,086, 2,979, 2,939, 1,921, 1,779, 1,740, 1,693, 1,612, 1,587, 1,568, 1,505, 1,456, 14,35, 1,401, 1,371, 1,354, 1,339, 1,325, 1,244, 1,227, 1,201, 1,170, 1,150, 1,095, 1,061, 1,044, 1,021, and 961.

### 6-((2-aminoethyl)amino)-2-ethyl-1H-benzo[de]isoquinoline-1,3(2H)-dione (2b)

6-bromo-2-ethyl-1H-benzo[de]isoquinoline-1,3(2H)-dione (1 g, 3.288 mMol, 1 eq) was stirred at room temperature overnight in neat ethylenediamine (10 mL, excess) to yield a dark orange liquid. The crude product was slowly added to deionized water (50 mL) and left to stir at room temperature for 2 h. The precipitate was collected by suction filtration and washed with H_2_O to yield the product as a yellow amorphous solid (0.748 g, 80%). ^**1**^**H NMR**, (DMSO-*d*_6_, 500.13 MHz) δ (ppm), *J* (Hz): 1.11 (t, *J* = 7.0, 3H, CH_3_), 2.82 (t, *J* = 6.4, 2H, CH_2_), 3.32 (t, *J* = 6.4, 2H, CH_2_), 3.97 (q, *J* = 7.1, 2H, CH_2_), 6.73 (d, *J* = 8.5, 1H, phenylene H), 7.60 (m, 1H, phenylene H), 8.18 (d, J = 8.5, 1H, phenylene H), 8.36 (dd, 1H, phenylene H), 8.63 (dd, 1H, phenylene H); ^**13**^**C NMR**, (DMSO-*d*_6_, 125.76 MHz) δ (ppm): 13.7, 34.7, 46.7, 104.3, 108.1, 120.6, 122.3, 124.6, 129.0, 129.8, 131.0, 134.6, 151.3, 163.1, 164.0; **HRMS** (ESI) calcd. for C_16_H_18_N_3_O_2_ [M + H]^+^ 284.139, found 284.1389; **ν**_max_ **(KBr)/cm**^**−1**^**:** 3,358, 2,979, 1,676, 1,612, 1,586, 1,550, 1,461, 1,430, 1,396, 1,370, 1,349, 1,249, 1,188, 1,152, 1,121, 1,104, 1,066, 965, and 918.

### Tert-butyl(2-aminoethyl)carbamate (3a)

To a 500 mL round bottom flask was added ethylenediamine (13.4 mL, 161.86 mMol, 10 eq) in CHCl_3_ (100 mL). A solution of di-tert-butyl dicarbonate (4.4 g, 37.56 mMol, 1 eq) in CHCl_3_ (50 mL) was added dropwise over 2 h at 0°C and stirred at room for 24 h. The reaction mixture was washed with brine, the organic layer was washed with de-ionized H_2_O and dried over MgSO_4_. The filtrate was concentrated in vacuo to yield an off-white liquid. (2.012 g, 34%). ^**1**^**H NMR**, (DMSO-*d*_6_, 500 MHz) δ (ppm), *J* (Hz): 1.36 (s, 9H, CH_3_), 2.51 (d, *J* = 6.5, 2H, CH_2_), 2.90 (d, *J* = 5.8, 2H, CH_2_), 6.71 (s, 1H, NH); ^**13**^**C NMR** (DMSO-*d*_6_, 125.76 MHz): δ (ppm): 28.7, 31.7, 42.0, 44.1, 77.8, 79.6, 156.1.

### Tert-butyl(2-(6-nitro-1,3-dioxo-1H-benzo[de]isoquinolin-2(3H)-yl)ethyl)carbamate (3b)

Tert-butyl (2-aminoethyl) carbamate (0.565 mL, 3.54 mMol, 1 eq) was slowly added dropwise to a solution of 4-Nitro-1,8-naphthalic anhydride (0.86 g, 3.54 mMol, 1 eq) in EtOH (20 mL). The reaction mixture was left to react in a 35 mL microwave tube for 1 h at 110°C, 1 mbar and 300 watts. The precipitate was collected by suction filtration and washed with EtOH and Et_2_O to yield the product as a peach amorphous solid (1.047 g, 76%). ^**1**^**H NMR**, (DMSO-*d*_6_, 500.13 MHz) δ (ppm), *J* (Hz): 1.22 (s, 9H, CH_3_), 3.27 (t, *J* = 6.0, 2H, CH_2_), 4.14 (t, J = 6.0, 2H, CH_2_), 6.88 (t, *J* = 6.3, 1H, NH), 8.09 (t, *J* = 8.1, 1H, phenylene H), 8.55 (d, *J* = 8.0, 1H, phenylene H), 8.62 (m, 2H, phenylene H), 8.71 (d, *J* = 8.4, 1H, phenylene H); ^**13**^**C NMR** (DMSO-*d*_6_, 125.76 MHz): δ (ppm): 28.5, 38.0, 77.9, 123.1, 123.5, 124.6, 127.3, 128.9, 129.0, 129.9, 130.5, 132.0, 149.5, 156.2, 162.8, 163.6; **HRMS** (ESI) calcd. for C_19_H_19_N_3_NaO_6_ [M + Na]^+^ 408.117, found 408.1144; **ν**_max_ **(KBr)/cm**^**−1**^**:** 3,401, 3,070, 2,977, 2,935, 1,714, 1,702, 1,623, 1,593, 1,584, 1,523, 1,447, 1,410, 1,365, 1,341, 1,272, 1,233, 1,255, 1,192, 1,169, 1,148, 1,064, 992, and 968.

### 2-(2-aminoethyl)-6-nitro-1H-benzo[de]isoquinoline-1,3(2H)-dione (3c)

Tert-butyl (2-(6-nitro-1,3-dioxo-1H-benzo[de]isoquinolin-2(3H)-yl)ethyl)carbamate (1.02 g, 2.647 mMol, 1 eq) was dissolved in (TFA: DCM, 50: 50) (6 mL) and was stirred at room temperature overnight. The solvent was removed under reduced pressure to yield a beige amorphous solid (0.70 g, 93%). ^**1**^**H NMR**, (DMSO-d_6_, 500 MHz) δ (ppm), *J* (Hz): 3.17 (q, *J* = 5.6, 2H, CH_2_), 4.33 (t, *J* = 5.6, 2H, CH_2_), 7.81 (br, 2H, NH_2_), 8.13 (m, 1H, phenylene H), 8.58 (d, *J* = 7.9, 1H, phenylene H), 8.66 (m, 2H, phenylene H), 8.76 (dd, 1H, phenylene H); ^**13**^**C NMR** (DMSO-d_6_, 125 MHz): δ (ppm): 37.9, 38.2, 123.2, 123.4, 124.7, 127.2, 128.9, 129.4, 130.1, 130.6, 132.2, 149.7, 163.3, 164.0; **HRMS** (ESI) calcd. for C_14_H_12_N_3_O_4_ [M + H]^+^ 286.082, found 286.0800; **ν**_max_ **(KBr)/cm**^**−1**^**:** 3,078, 1,712, 1,595, 1,525, 1,465, 1,437, 1,384, 1,348, 1,247, 1,151, 1,093, 1,058, 1,020, and 981.

### 6-amino-2-(2-aminoethyl)-1H-benzo[de]isoquinoline-1,3(2H)-dione (3d)

Pd/C (~0.2 g) was added to a solution of 2-(2-aminoethyl)-6-nitro-1H-benzo[de]isoquinoline-1,3(2H)-dione (1.00 g, 3.50 mMol) dissolved in MeOH (40 mL). The reaction was placed under a H_2_ atmosphere and left to stir at room temperature for 3 h. The reaction was filtered through a pad of celite and washed with excess MeOH, the filtrate was removed under reduced pressure to yield a mustard amorphous solid (0.673g, 75%). ^**1**^**H NMR**, (DMSO-*d*_6_, 500.13 MHz) δ (ppm), *J* (Hz): 3.12 (s, 2H, CH_2_), 4.27 (t, *J* = 6.0, 2H, CH_2_), 6.86 (d, *J* = 8.4, 1H, phenylene H), 7.48 (s, 2H, NH_2_), 7.67 (m, 1H, phenylene H), 7.77 (br, 2H, NH_2_), 8.21 (d, J = 8.4, 1H, phenylene H), 8.44 (dd, 1H, phenylene H), 8.63 (dd, 1H, phenylene H); ^**13**^**C NMR**, (DMSO-*d*_6_, 125.76 MHz) δ (ppm): 37.62, 38.36, 108.01, 108.63, 119.84, 122.43, 124.46, 129.94, 130.46, 131.52, 134.52, 153.40, 163.89, 164.96; **HRMS** (ESI) calcd. for C_14_H_14_N_3_O_2_ [M + H]^+^ 256.108, found 256.1055; **ν**_max_ **(KBr)/cm**^**−1**^**:** 3,419, 3,362, 3,259, 3,017, 1,636, 1,582, 1,531, 1,485, 1,429, 1,402, 1,378, 1,353, 1,311, 1,247, 1,203, 1,172, 1,128, 1,016, 964, and 907.

### 6-((2-((2-((3,5-Bis(Trifluoromethyl)Phenyl)Amino)-3,4-Dioxocyclobut-1-en-1-yl)Amino)Ethyl)Amino)-2-Ethyl-1H-Benzo[de]Isoquinoline−1,3-(2H)-Dione (SQ-1)

A solution of 3-((3,5-bis(trifluoromethyl)phenyl)amino)-4-ethoxycyclobut-3-ene-1,2-dione (0.436 g, 1.235 mMol, 1 eq) in EtOH (8 mL) was added slowly to a mixture of 6-((2-aminoethyl)amino)-2-ethyl-1H-benzo[de]isoquinoline-1,3(2H)-dione (0.35 g, 1.235 mM, 1 eq) and Et_3_N (0.689 mL, 4.94 mMol, 4 eq) in EtOH (12 mL). The reaction was stirred at room temperature overnight. The precipitate was collected by suction filtration and washed with EtOH and ether. Product as a yellow amorphous solid with 78% yield (0.575 g). ^1^H NMR at 343K, (DMSO- *d*_6_, 500 MHz) δ (ppm), *J* (Hz): 1.18 (t, *J* = 7.0, 3H, CH_3_), 3.70 (q, *J* = 5.5, 2H, CH_2_), 3.95 (t, *J* = 5.5, 2H, CH_2_), 4.04 (q, *J* = 7.0, 2H, CH_2_), 6.91 (d, *J* = 8.5, 1H, phenylene H), 7.47 (s, 1H, phenylene H), 7.63 (m, 2H, phenylene H), 7.80 (br, 2H, NH), 7.89 (s, 1H, phenylene H), 8.25 (d, *J* = 8.5, 1H, phenylene H), 8.39 (d, J = 7.0, 1H, phenylene H), 8.58 (d, *J* = 8.3, 1H, phenylene H), 9.68 (br, 1H, NH); ^13^C NMR at 343K, (DMSO-d_6_, 125.76 MHz) δ (ppm): 13.6, 30.9, 34.6, 43.2, 44.2, 104.6, 109.1, 114.7, 118.3, 120.8, 122.5, 124.7, 128.6, 129.8, 130.9, 134.2, 141.5, 150.7, 163.1, 163.9, 171.2, 181.3; HRMS (ESI) calcd. for C_28_H_21_F_6_N_4_O_4_ [M + H]^+^ 591.146 found 591.1439; ν_max_ (KBr)/cm-1: 3,292 (broad), 3,086, 2,982, 1,796, 1,687, 1,581, 1,548, 1,459, 1,348, 1,278, 1,249, 1,180, 1,130, 1,067, 999, and 932.

### 6-amino-2-(2-((2-((3,5-bis(trifluoromethyl)phenyl)amino)-3,4-dioxocyclobut-1-en-1-yl)amino)ethyl)-1H-benzo[de]isoquinoline-1,3(2H)-dione (SQ-2)

A solution of 3-((3,5-bis(trifluoromethyl)phenyl)amino)-4-ethoxycyclobut-3-ene-1,2-dione (0.14 g, 0.39 mMol, 1 eq) in EtOH (8 mL) was slowly added to a mixture of 6-amino-2-(2-aminoethyl)-1H-benzo[de]isoquinoline-1,3(2H)-dione (0.1 g, 0.39 mmol, 1 eq) and Et3N (0.217 mL, 1.56 mMol, 4 eq) in EtOH (10 mL). The reaction was stirred at room temperature overnight. The precipitate was collected by suction filtration and washed with EtOH and Et2O to yield the product as an olive green amorphous solid (0.123 g, 56%). ^1^H NMR at 343K, (DMSO-d_6_, 500.13 MHz) δ (ppm), *J* (Hz): 3.90 (s, 2H, CH_2_), 4.31 (t, *J* = 6.0, 2H, CH_2_), 6.82 (d, *J* = 8.4, 1H, phenylene H), 7.22 (s, 2H, NH_2_), 7.52 (s, 1H, phenylene H), 7.58 (t, *J* = 8.0, 1H, phenylene H), 7.89 (s, 2H, phenylene H), 8.15 (d, *J* = 8.4, 1H, phenylene H), 8.37 (d, *J* = 7.1, 1H, phenylene H), 8.57 (d, *J* = 8.4, 1H, phenylene H), 9.77 (br, 1H, NH); ^13^C NMR at 343K, (DMSO-d_6_, 125.76 MHz) δ (ppm): 27.9, 42.9, 108.7, 114.8, 118.4, 119.9, 120.3, 122.2, 122.5, 124.2, 124.7, 129.0, 130.3, 131.3, 134.3, 141.7, 153.3, 163.3, 164.6, 171.4, 181.3; HRMS (ESI) calcd. for C_52_H_33_F_12_N_8_O_8_ [2M + H]^+^ 1125.223, found 1125.2226; ν_max_ (KBr)/cm^−1^: 371, 3,254, 1,796, 1,692, 1,636, 1,599, 1,528, 1,476, 1,434, 1,378, 1,308, 1,276, 1,250, 1,184, 1,129, 1,068, 1,000, and 933.

## Data Availability

The datasets generated for this study are available on request to the corresponding author.

## Author Contributions

RE, LK, and MS designed the study and wrote the manuscript. RE and MS supervised the study. AA and LK synthesized and characterized the compounds and carried out all spectroscopic titrations. OF performed the SEM studies. MS, MK, and JP carried out the biological studies. All authors discussed the results and commented on the manuscript.

### Conflict of Interest Statement

The authors declare that the research was conducted in the absence of any commercial or financial relationships that could be construed as a potential conflict of interest.
