# Supplementary material for: Squaramide—Naphthalimide Conjugates as “Turn-On” Fluorescent Sensors for Bromide Through an Aggregation-Disaggregation Approach
Source: Front Chem. 2019 May 22;7:354. doi: 10.3389/fchem.2019.00354 (PMC6540876; doi:10.3389/fchem.2019.00354)
Supplement: Supplementary file 1 [file Data_Sheet_1.docx]

**Squaramide – Naphthalimide Conjugates as ‘Turn-On’ Fluorescent Sensors for Bromide Through an Aggregation-Disaggregation Approach.**

Lokesh K. Kumawat^1^, Anthony A. Abogunrin^1^, Michelle Kickham^1,2^, Jyotsna Pardeshi^2^, Orla Fenelon^1^, Martina Schroeder^2^ and Robert B. P. Elmes^1^*

^1^Department of Chemistry, Maynooth University, National University of Ireland, Maynooth, Co. Kildare, Ireland.

Tel: +353 1 7084615; E-mail: [robert.elmes@mu.ie](mailto:robert.elmes@mu.ie)

^2^Department of Biology, Maynooth University, National University of Ireland, Maynooth, Co. Kildare, Ireland.

**Supporting Information**

| **1** | Materials and Methods | **Page** |
| --- | --- | --- |
| **2** | Reaction Scheme | **4** |
| **3** | ^1^H & ^13^C NMR spectra of all compounds | **5-22** |
| **4** | Disaggregation behaviour of probes at different temperature (NMR and Fluorescence Study) | **23-26** |
| **5** | Qualitative NMR Anion Titrations. | **27** |
| **6** | Fluorescence response of **SQ1** and **SQ2** with all anions | **28-32** |
| **7** | Association constant | **33-36** |
| **8** | NMR Titration | **37-40** |
| **9** | Biological Data (Cytotoxicity, Confocal Microscopy) | **40-42** |

***Materials and Methods:***

Commercial materials were supplied by TCI Europe or Sigma Aldrich and were used without further purification. HPLC grade solvents were used as received. ^1^H NMR spectra were recorded using a Bruker Avance III 500 at a frequency of 500.13 MHz, and are reported as parts per million (ppm) with CDCl_3_ (δH 7.26 ppm) or DMSO-*d_6_* (δH 2.50 ppm) as an internal reference. The data are reported as chemical shift (δ), multiplicity (br = broad, s = singlet, d = doublet, t = triplet, m = multiplet), coupling constant (*J*, Hz) and relative integral. ^13^C NMR spectra were recorded using a Bruker Avance III 500 at a frequency of 125.76 MHz and are reported as parts per million (ppm) with CDCl_3_ (δH 77.1 ppm) or DMSO-*d_6_* (δH 39.5 ppm) as an internal reference. High resolution ESI spectra were recorded on an Agilent 6310 LCMS TOF. Analytical TLC was performed using pre-coated silica gel plates (Merck Kieselgel 60 F254). Flash chromatography was performed using silica gel 40-63 µM, 60 Å. Infrared absorption spectra were recorded on a Perkin Elmer Spectrum 100 FT-IR spectrometer using KBr disks. FT-IR are reported in wavenumbers (cm^-1^). Diethyl squarate were synthesised as previously described.[^1^](#_ENREF_1)

**Spectroscopic Binding Studies:** Spectroscopic titrations were performed by additions of aliquots of the putative anionic guest as the tetrabutylammonium (TBA) salt solution (20 mM) in DMSO to a solution of the receptor in DMSO. After each addition, the resulting solution was stirred for at least 30 seconds and the fluorescence was recorded. Both salt and receptor were dried under high vacuum prior to use. Fluorescence titrations were carried out using a Jasco FP-6300 spectrofluorometer.

**NMR Binding Studies:** NMR titrations were performed by additions of aliquots of the putative anionic guest as the tetrabutylammonium (TBA) salt, to a solution of the receptor in DMSO- *d_6_*. Typically, up to 30 equivalents of the anion were added to the solution. Both salt and receptor were dried under high vacuum prior to use. ^1^H NMR spectra were recorded on a Bruker Avance III 500 spectrometer at a frequency of 500.13 MHz and calibrated to the residual protio solvent peak in DMSO-*d_6_* (δ = 2.50 ppm). Stack plots were made using TopSpin 3.5. A global fitting analysis assuming a 1:1 binding model was employed to provide the binding constant (*K*_a_/M^-1^), by fitting of the chemical shift changes of the squaramide NH as a function of added anion using the open access BindFit software program.[^2-4^](#_ENREF_2)

**Confocal Microscopy:** 13mm cover slips (*VWR International*) were placed in the wells of a 24 well plate. HeLa cells were seeded at 0.5 x 10^5^ cells/ml and left to adhere to the cover slips overnight. 20µM, 5µM or 1µM of **SQ1** or **SQ2** (in DMSO) were added to the cells and incubated for 1 hour at 37°C and 5% CO_2_. Cells were then washed twice with PBS and fixed using 4% PFA. Cells were washed again with PBS and incubated with DAPI prior to mounting on microscope slides using ProLong Antifade Reagent (*Invitrogen)* and sealing with clear nail varnish. Slides were imaged using an Olympus Fluoview Confocal Microscope and analysed using Olympus Fluoview FV10-ASW software. Images were captured using a 40x objective lens. Sequential excitation at 405nm and 488nm was used.

**Flow Cytometry:** HeLa cells in suspension were incubated with 10µM, 1µM, 100nM, 20nM, 5nM or 1nM of SQ1 or SQ2 (in DMSO) with and without 50mM NaBr for one hour at 37°C with gentle agitation. Cells were then washed with PBS and resuspended in 100µL of PBS. Fluorescence intensity (FL-1) was analysed by flow cytometry using an Accuri C6 Flow Cytometer (BD Biosciences), after gating for viable cells on a FFS/SSC plot. Fluorescence intensity data was analysed using the software C-Flow Plus.

**MTT assay:** HeLa cells were seeded in a 96 well-plate at 0.75 x 10^5^ cells/ml and treated with different concentrations of **SQ1** or **SQ2** in triplicate. The plates were then incubated for 20h at 37°C and 5% CO_2_. Cell culture medium was then removed and MTT solution was added to each well (150ug/well), followed by incubation for 5h. MTT solution was then removed from the wells by aspiration. Formazan crystals formed as a result of metabolic activity of cells were dissolved by adding 200ul of DMSO and gently rocking the plate for 15 minutes at room temperature. Absorbance was measured at 550nm using a BMG Clariostar plate reader. The data was analyzed by subtracting media blank absorbance values from sample absorbance values. Percent viability was calculated with the following equation: %viable cells= (Abs sample-AbsBlank)/(Abs untr-AbsBlank) *100.

**Reaction Scheme:**

**Scheme S1:** Synthesis of **SQ1** and **SQ2**.


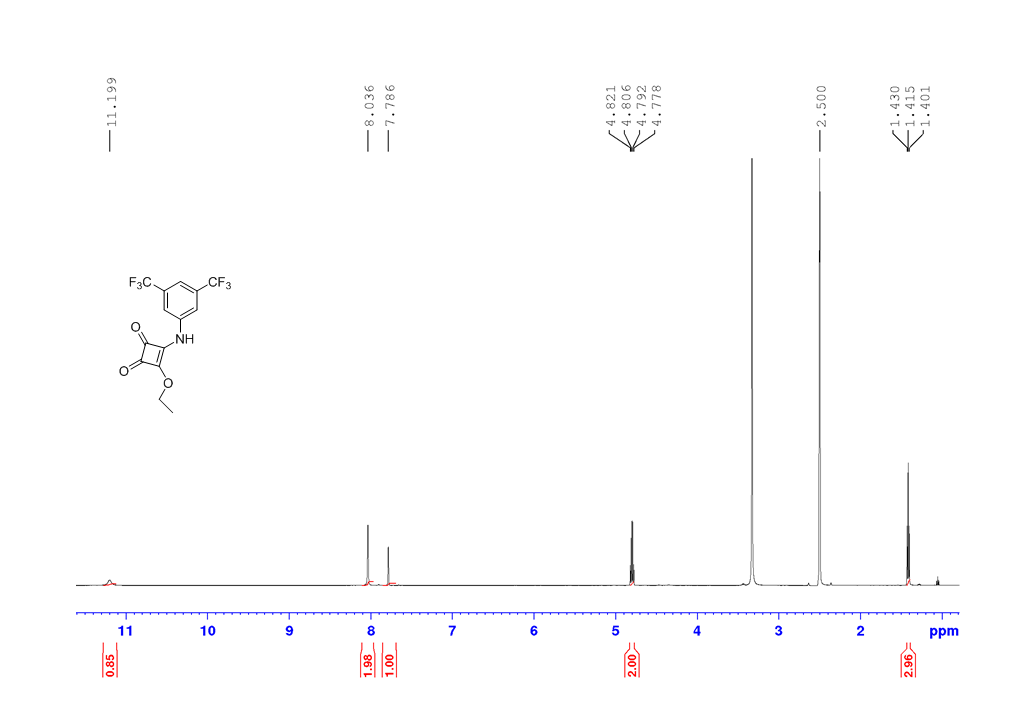


***Figure S1:*** *The ^1^H NMR spectra of* ***1*** *at 298K (500 MHz, DMSO-_d6_)*


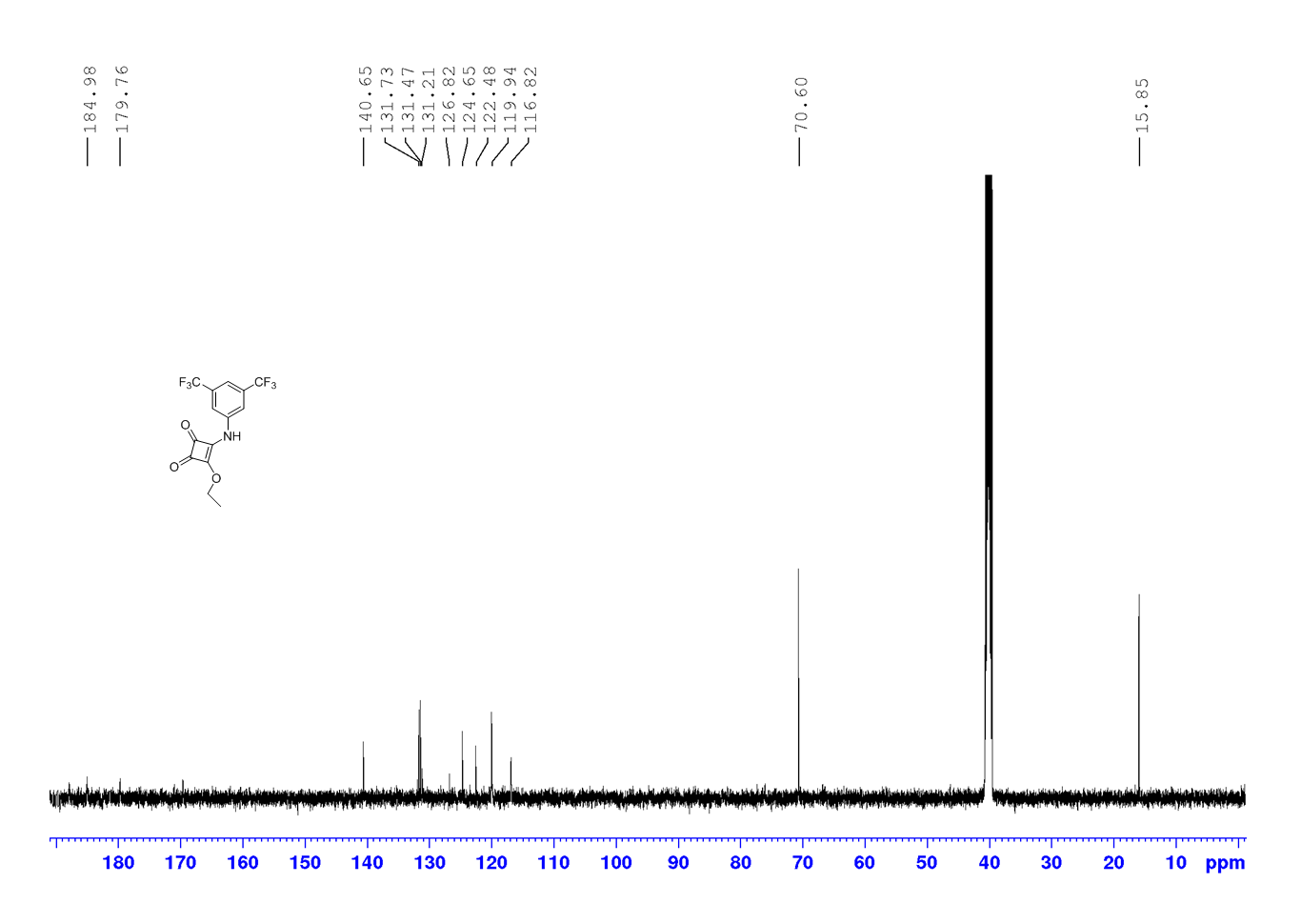


***Figure S2:*** *The ^13^C NMR spectra of* ***1*** *at 298K (125 MHz, DMSO-_d6_)*


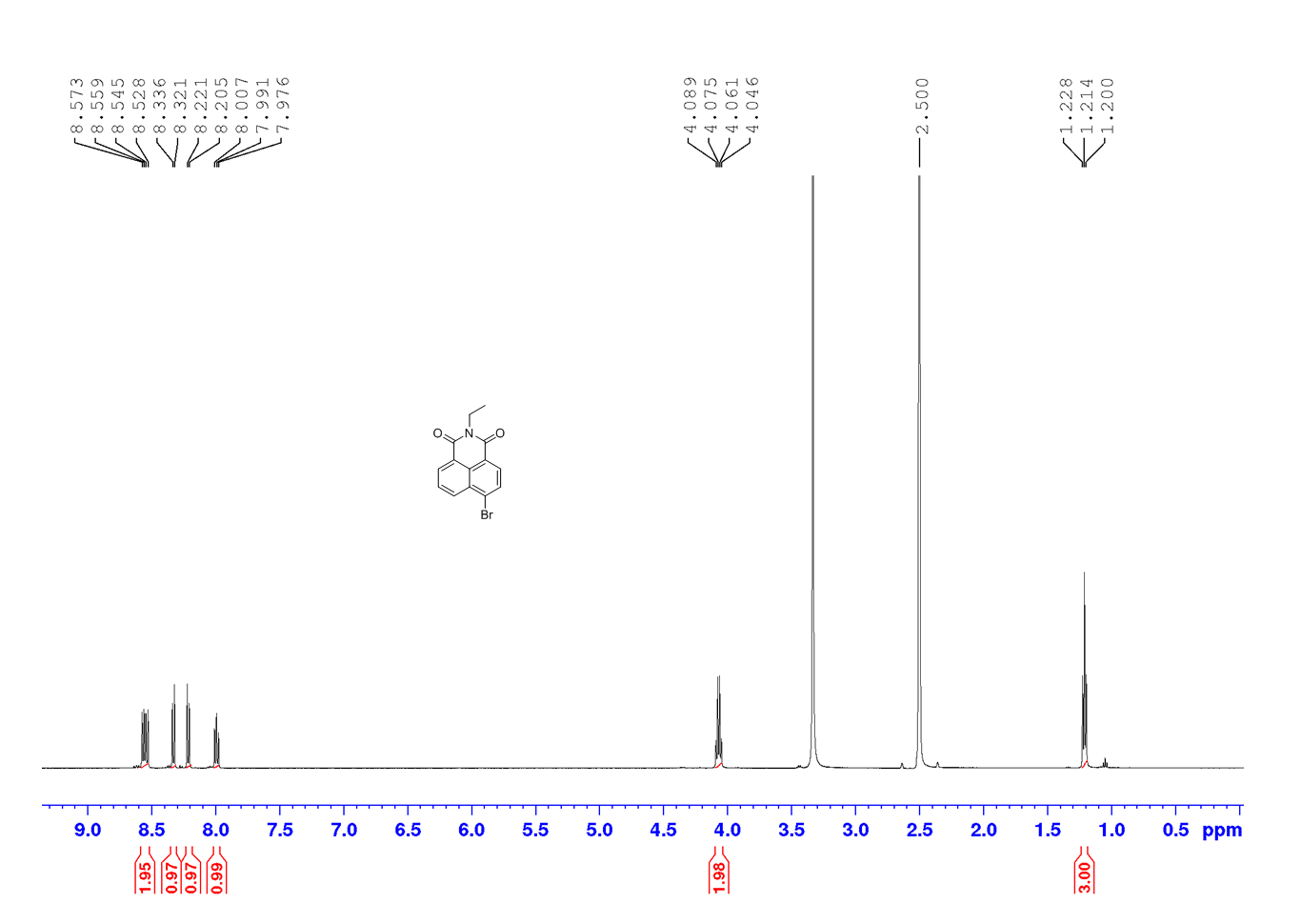


***Figure S3:*** *The ^1^H NMR spectra of* ***2a*** *at 298K (500 MHz, DMSO-_d6_)*


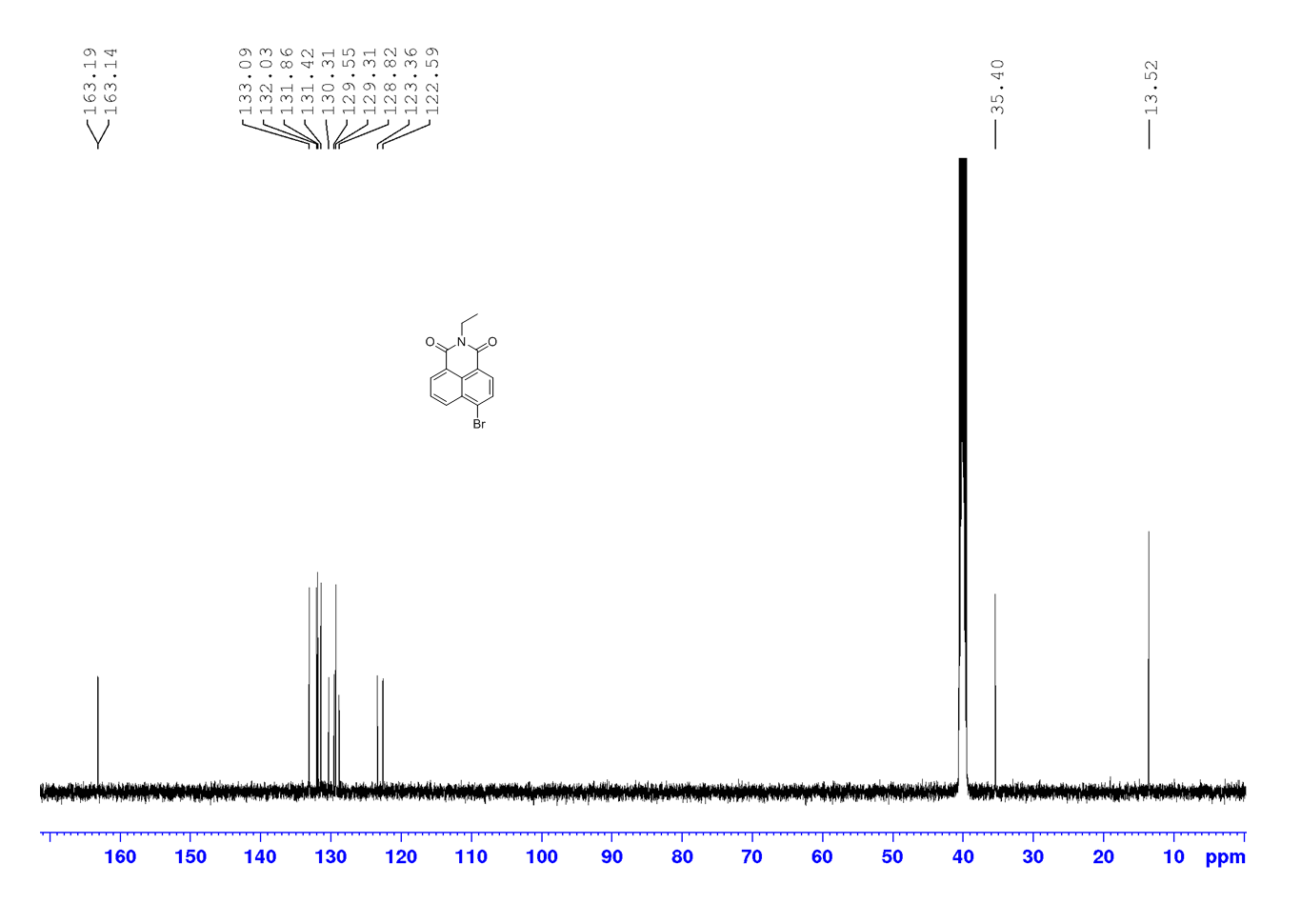


***Figure S4:*** *The ^13^C NMR spectra of* ***2a*** *at 298K (125 MHz, DMSO-_d6_)*


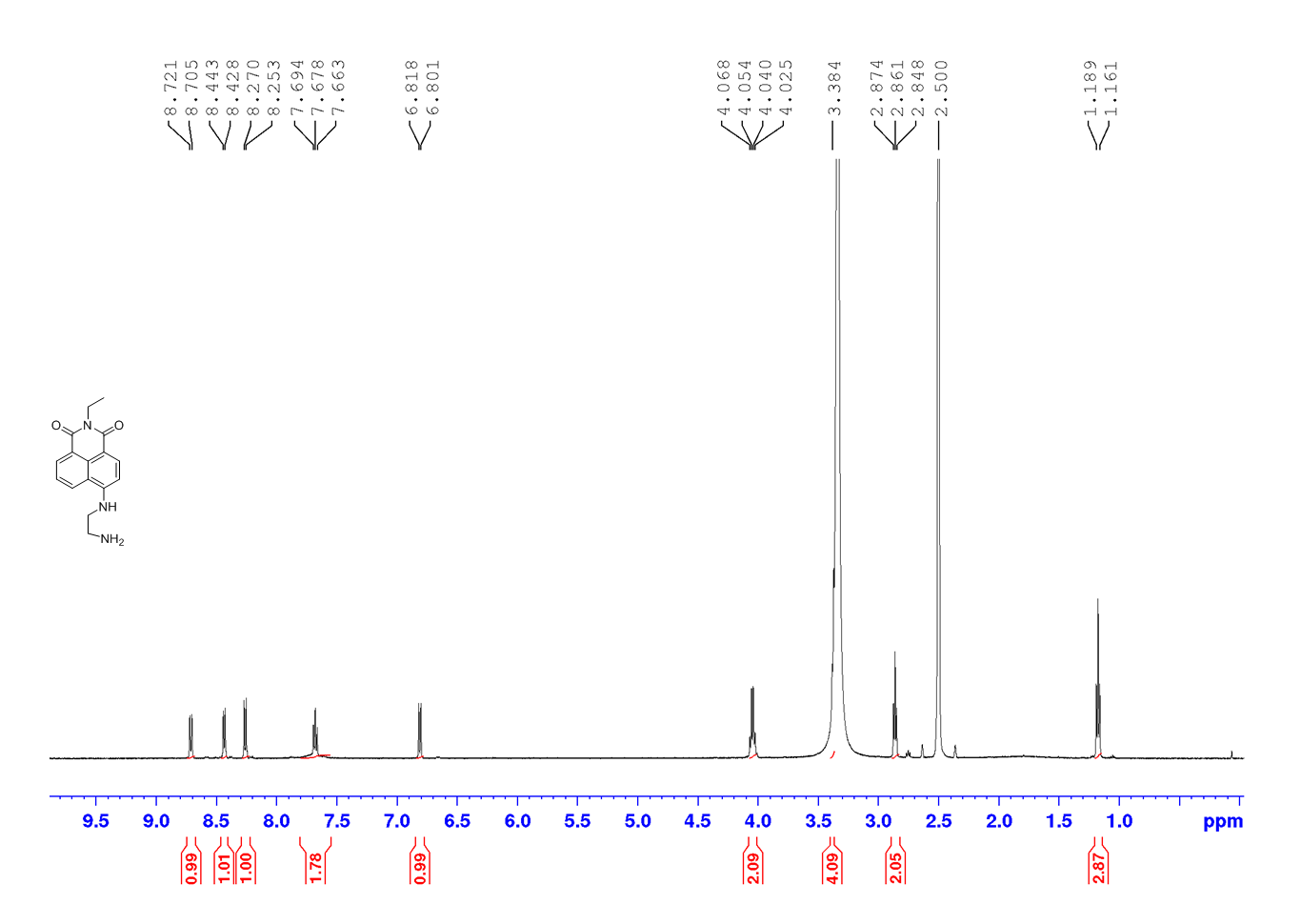


***Figure S5:*** *The ^1^H NMR spectra of* ***2b*** *at 298K (500 MHz, DMSO-_d6_)*


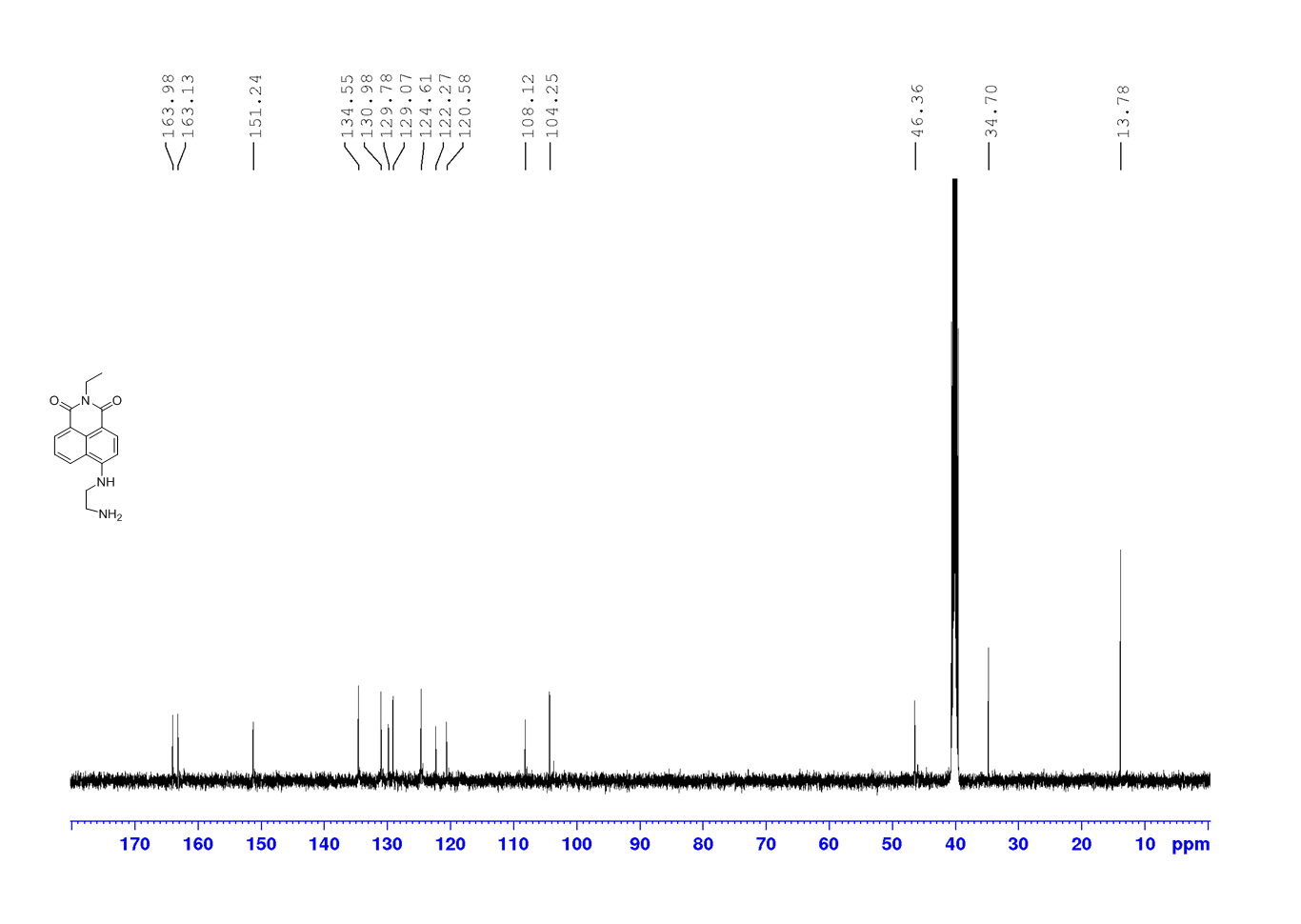


***Figure S6:*** *The ^13^C NMR spectra of* ***2b*** *at 298K (125 MHz, DMSO-_d6_)*

*
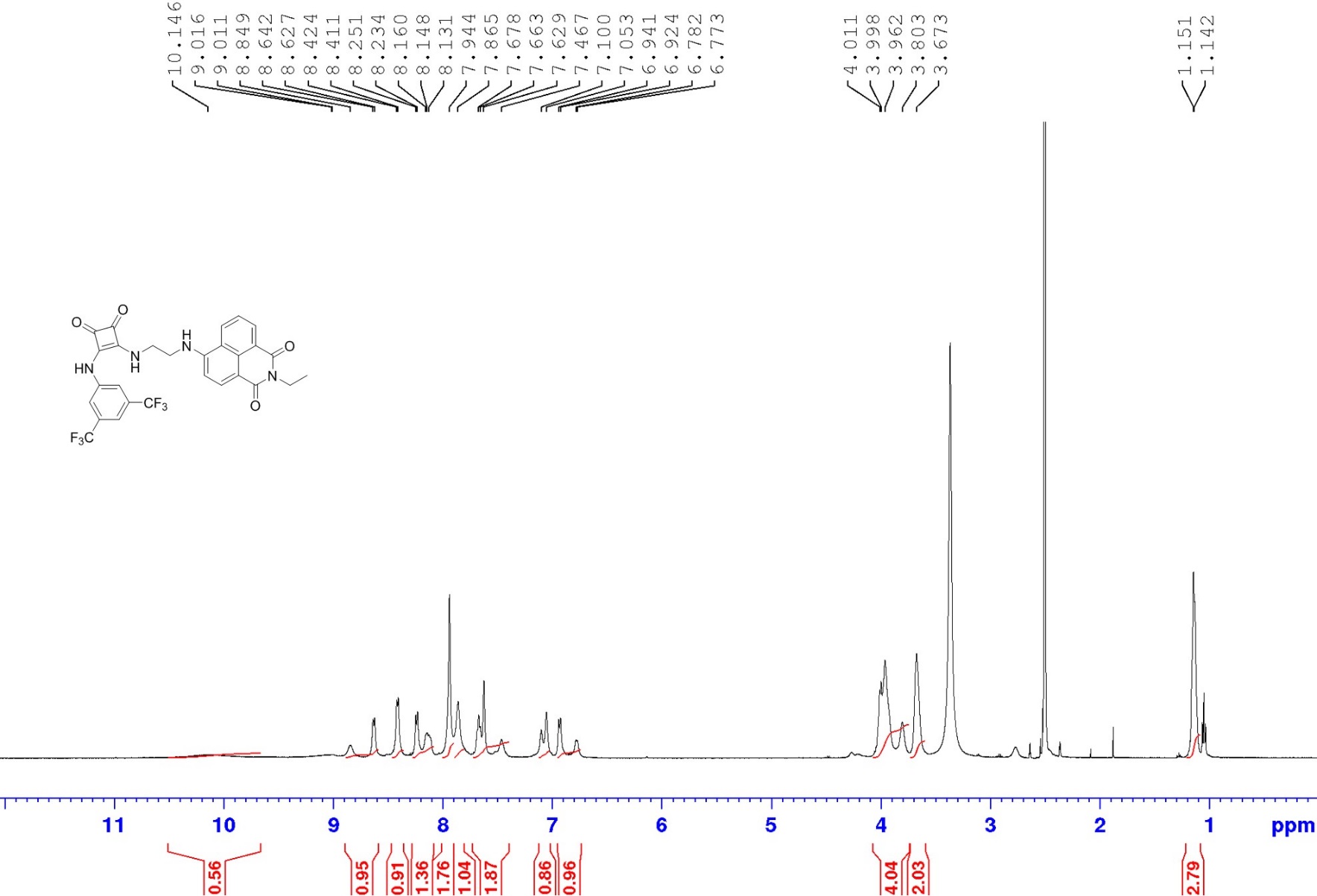
*

***Figure S7:*** *The ^1^H NMR spectra of* ***SQ1*** *at 298K (500 MHz, DMSO-_d6_)*

*
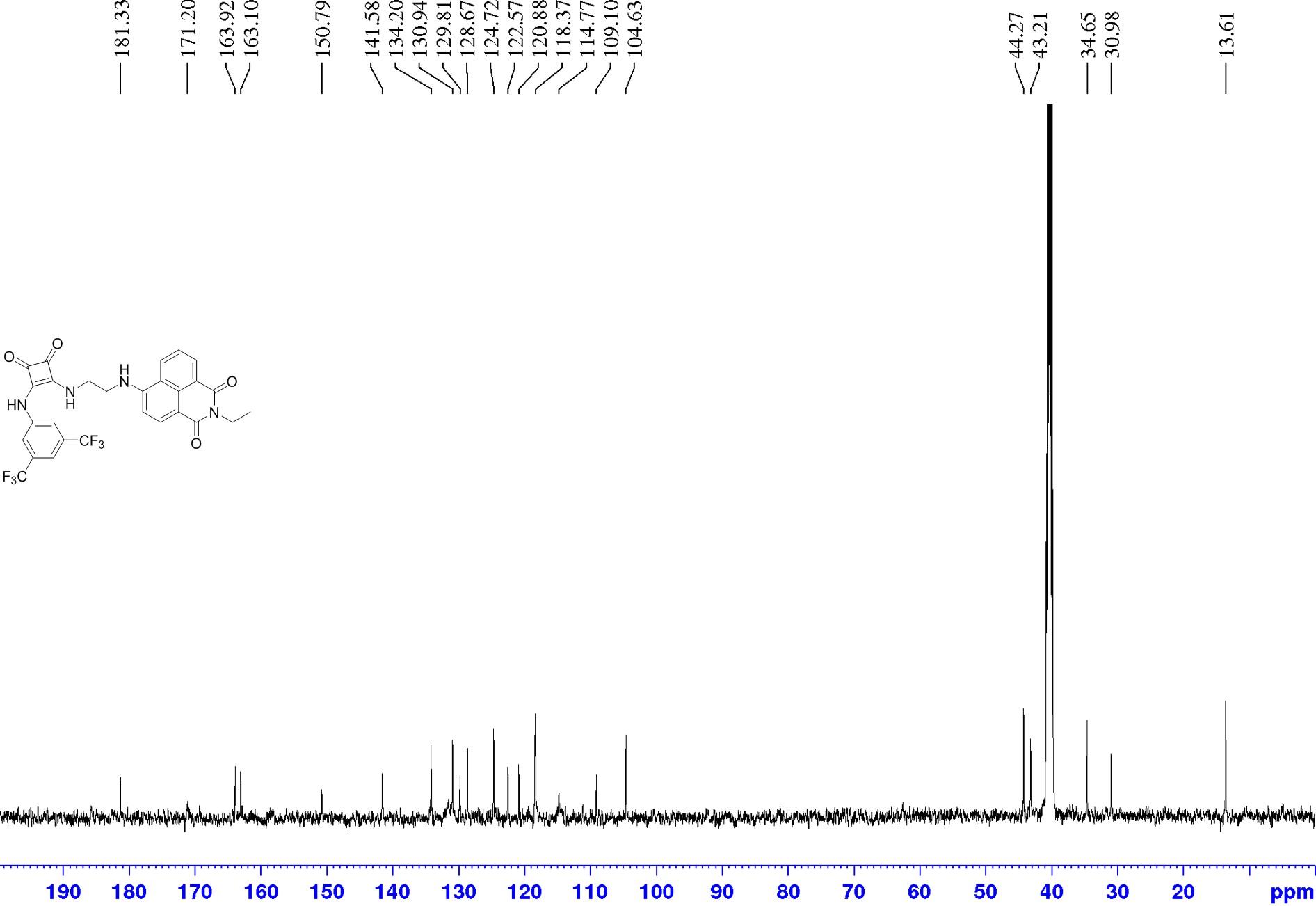
*

***Figure S8:*** *The ^13^C NMR spectra of* ***SQ1*** *at 343K (125 MHz, DMSO-_d6_)*


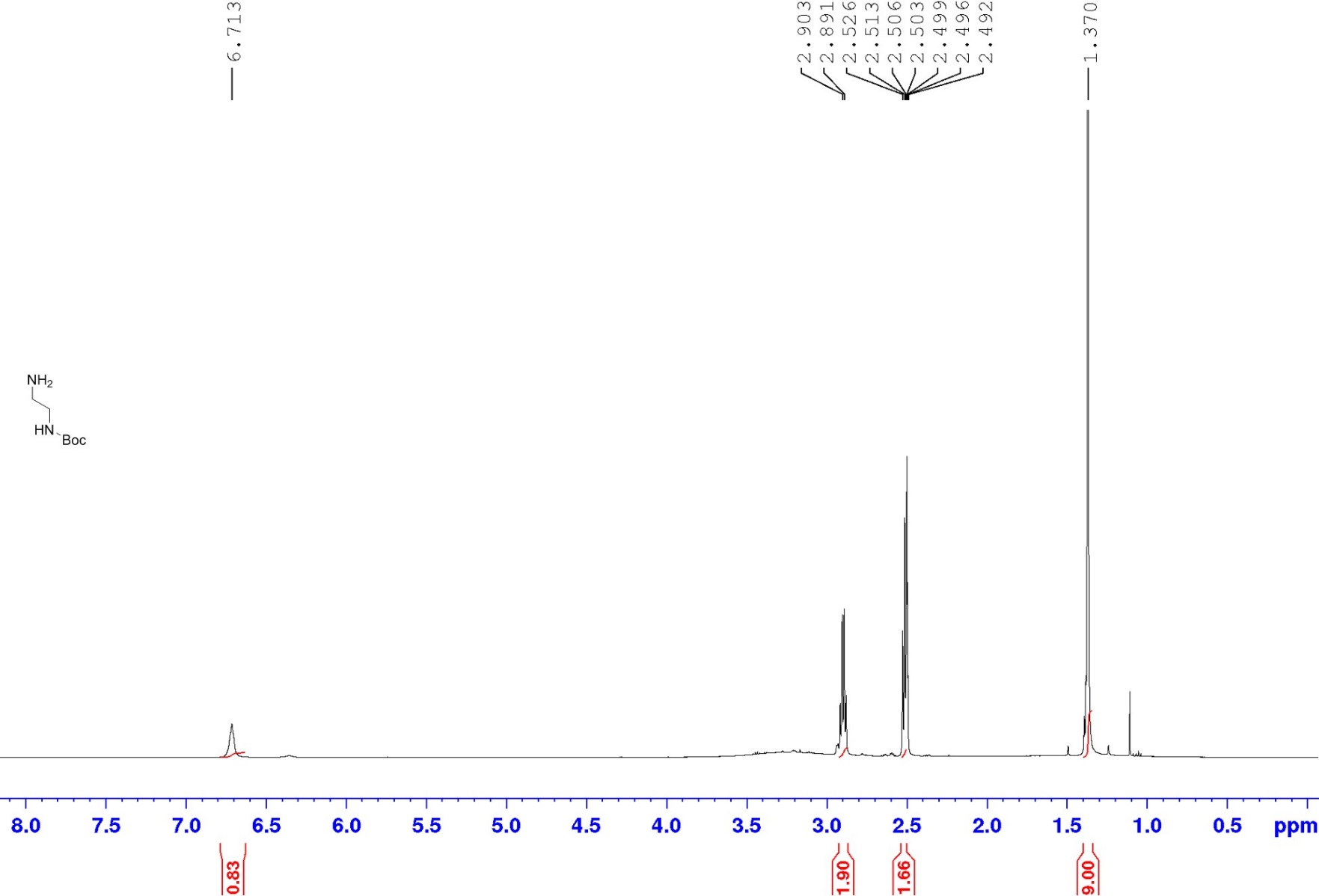


***Figure S9:*** *The ^1^H NMR spectra of* ***3a*** *at 298K (500 MHz, CDCl_3_)*


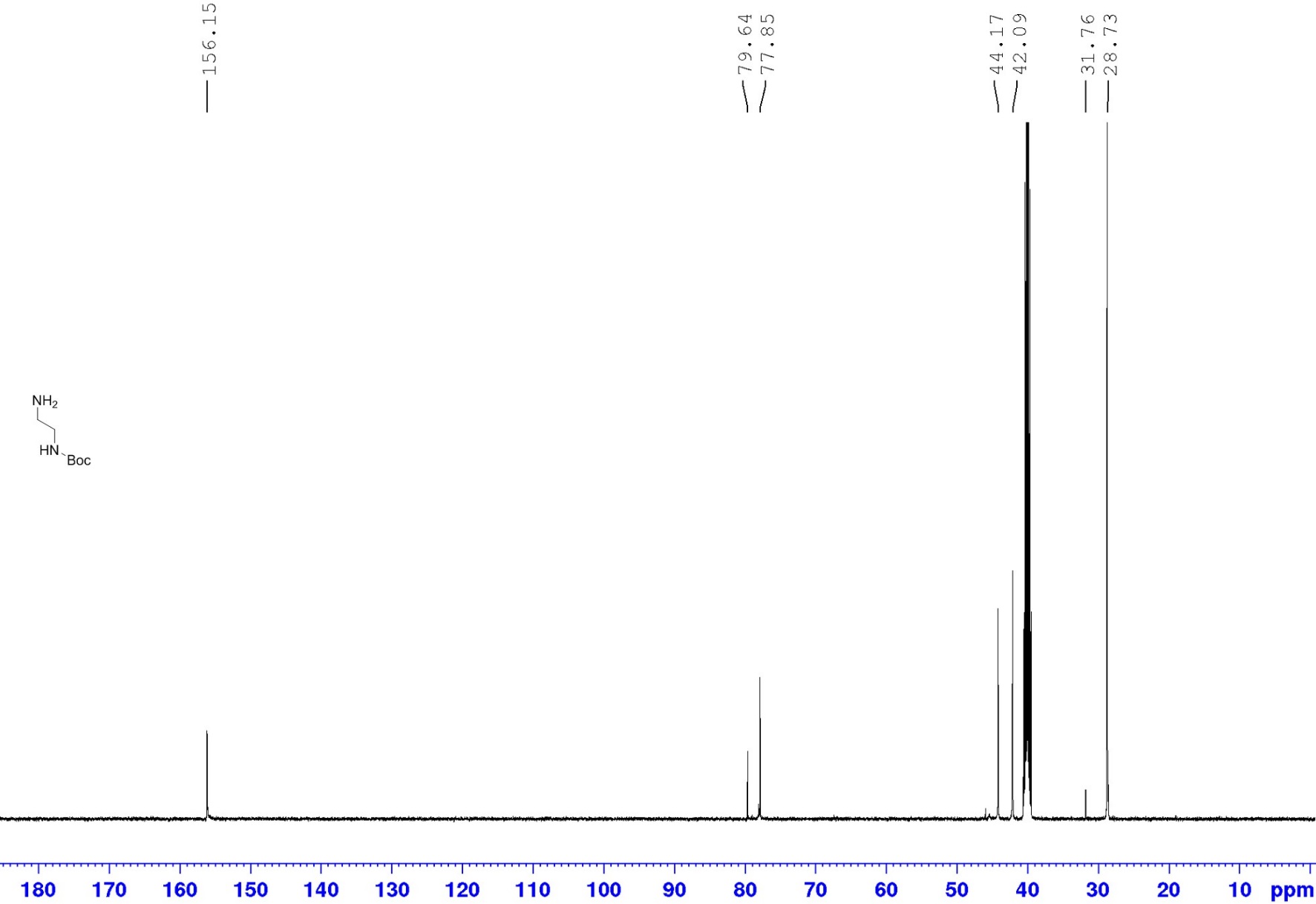


***Figure S10:*** *The ^13^C NMR spectra of* ***3a*** *at 298K (125 MHz, CDCl_3_)*


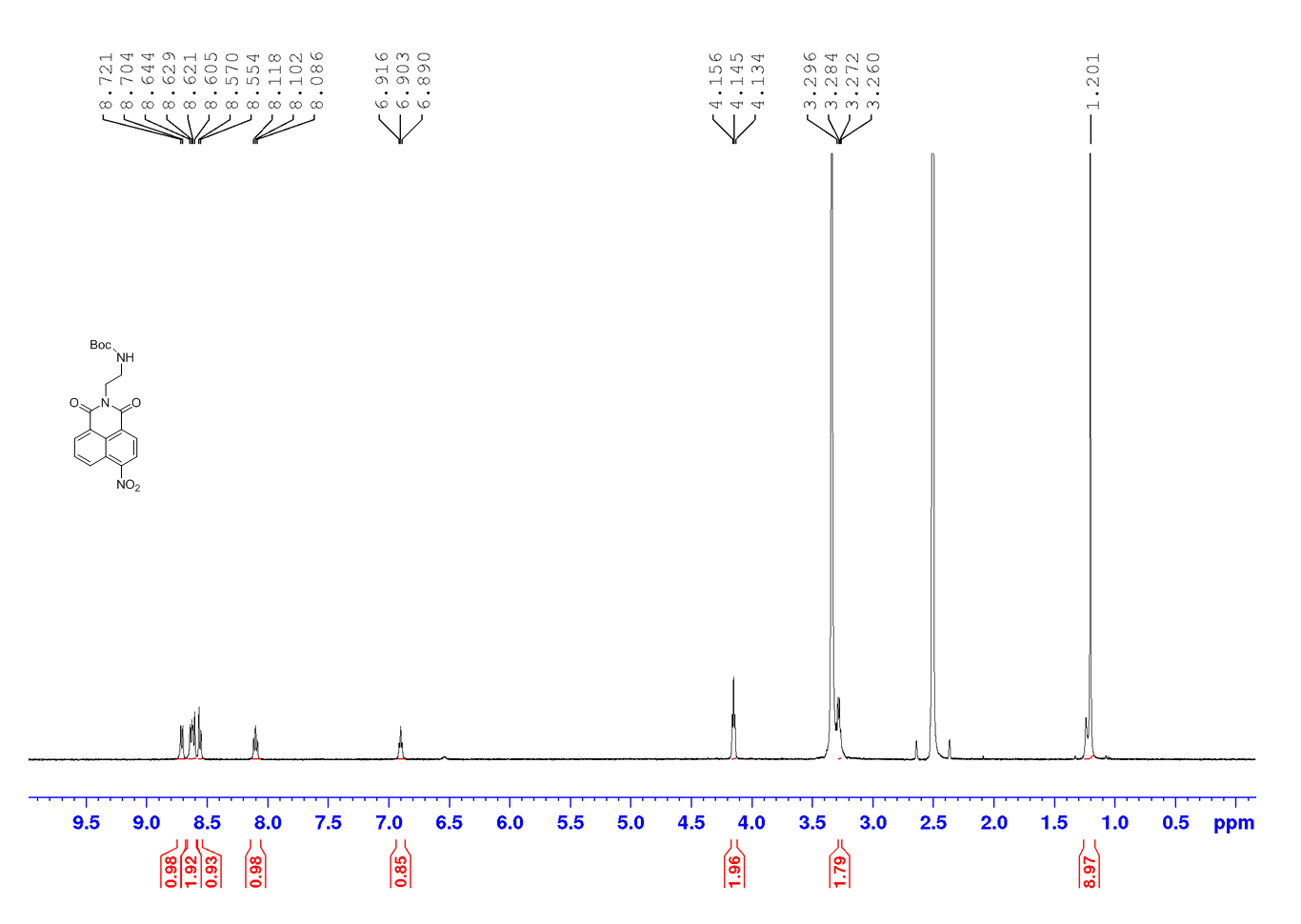


***Figure S11:*** *The ^1^H NMR spectra of* ***3b*** *at 298K (500 MHz, DMSO-_d6_)*


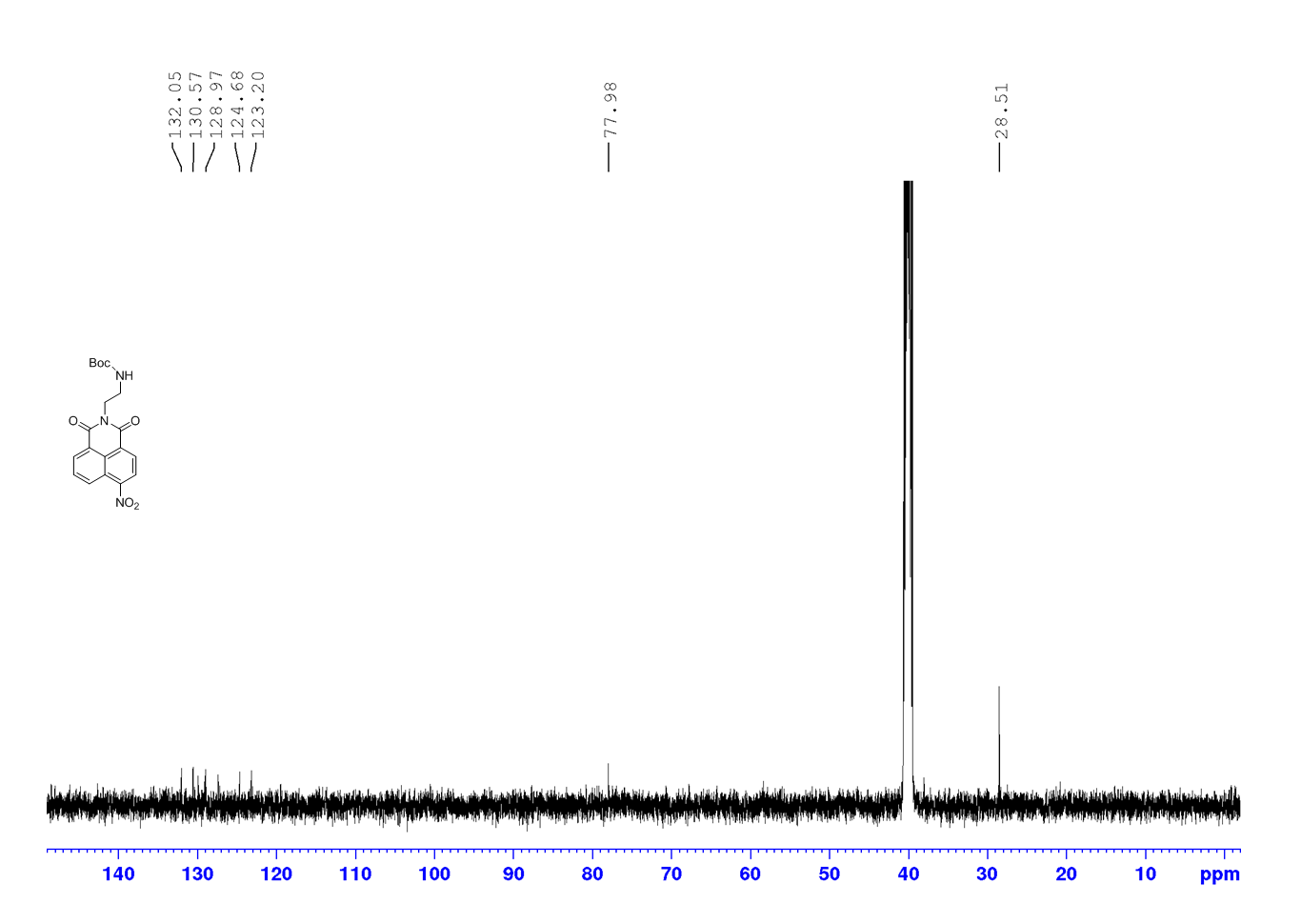


***Figure S12:*** *The ^13^C NMR spectra of* ***3b*** *at 298K (125 MHz, DMSO-_d6_)*


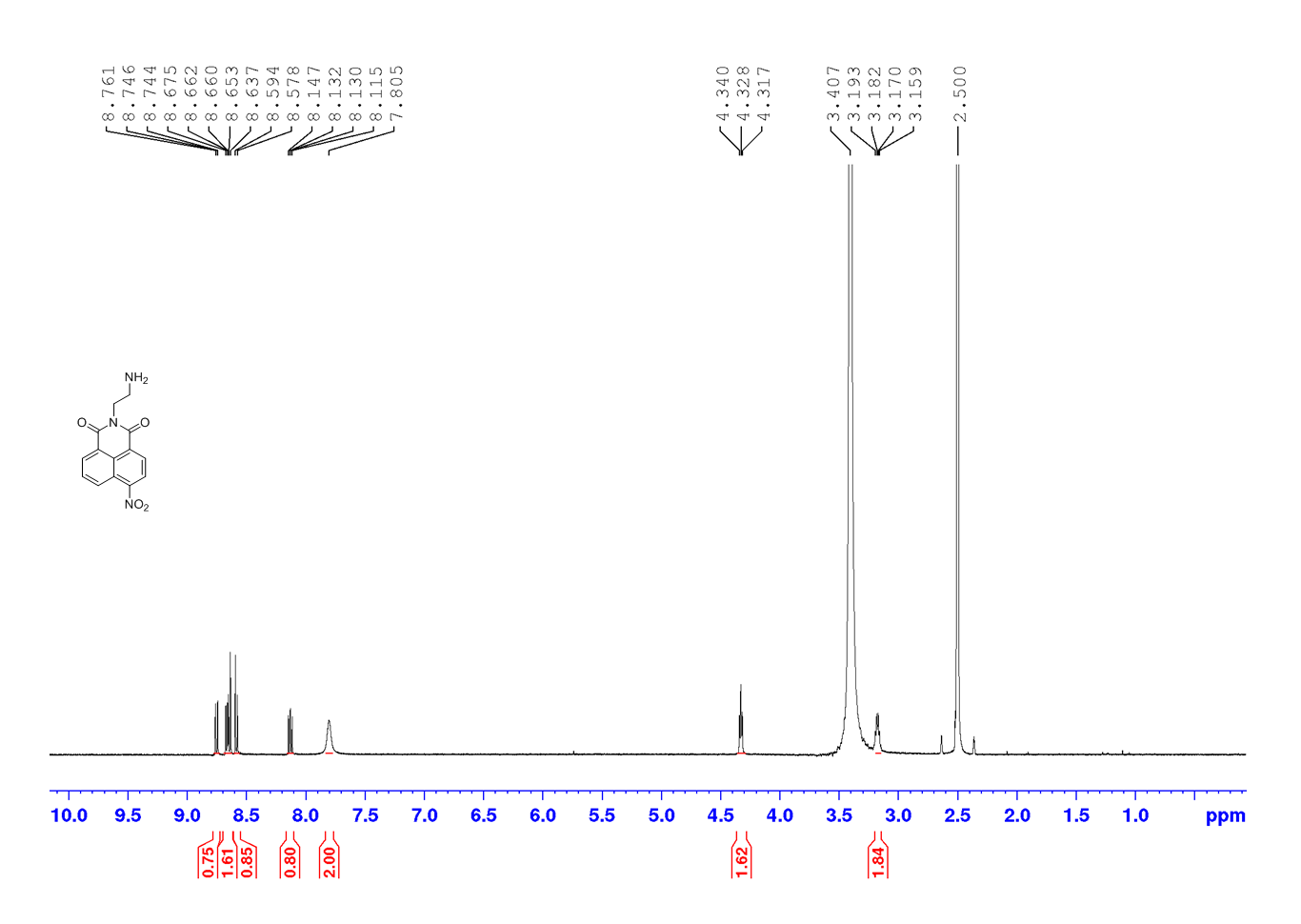


***Figure S13:*** *The ^1^H NMR spectra of* ***3c*** *at 298K (500 MHz, DMSO-_d6_)*


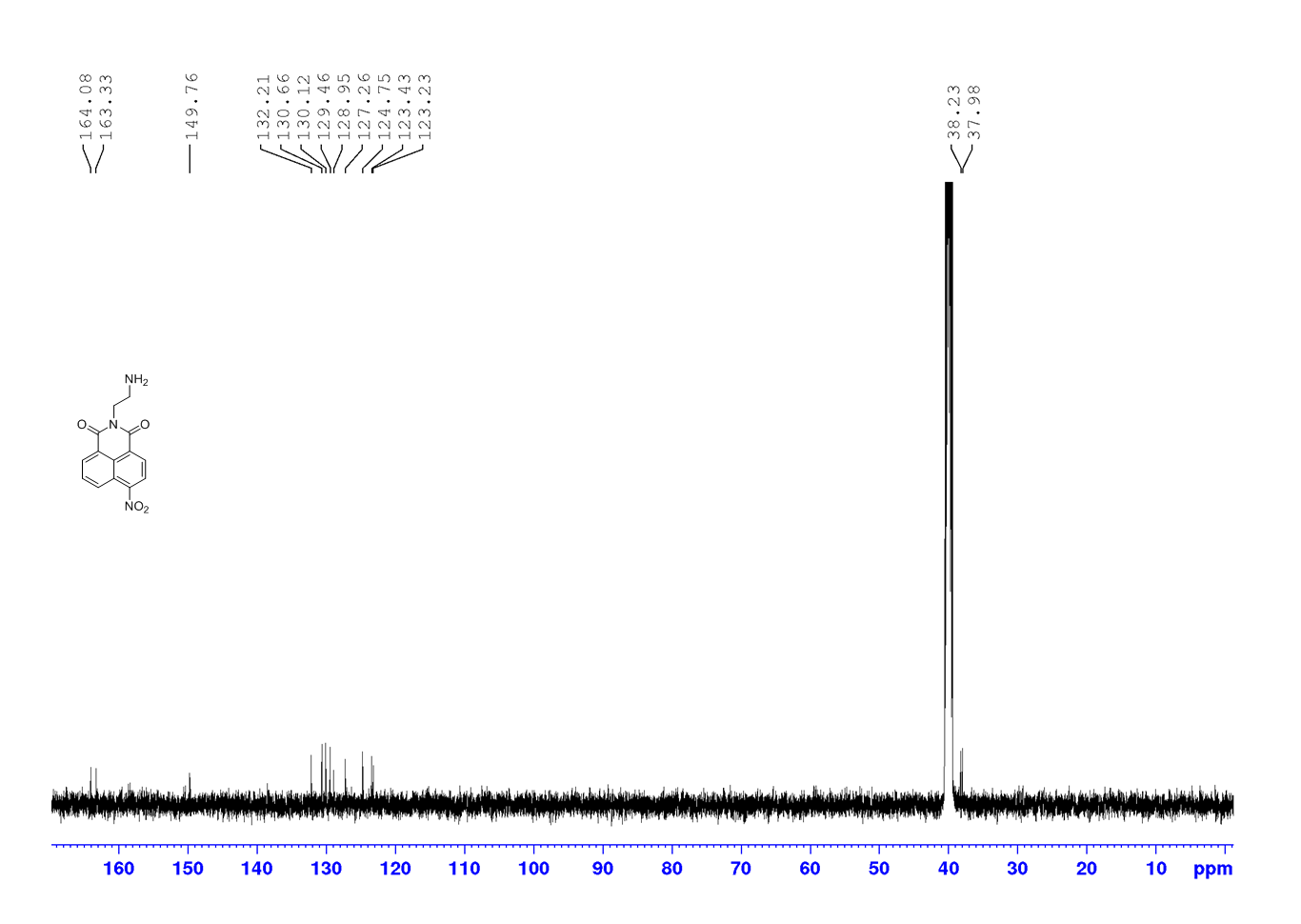


***Figure S14:*** *The ^13^C NMR spectra of* ***3c*** *at 298K (125 MHz, DMSO-_d6_)*


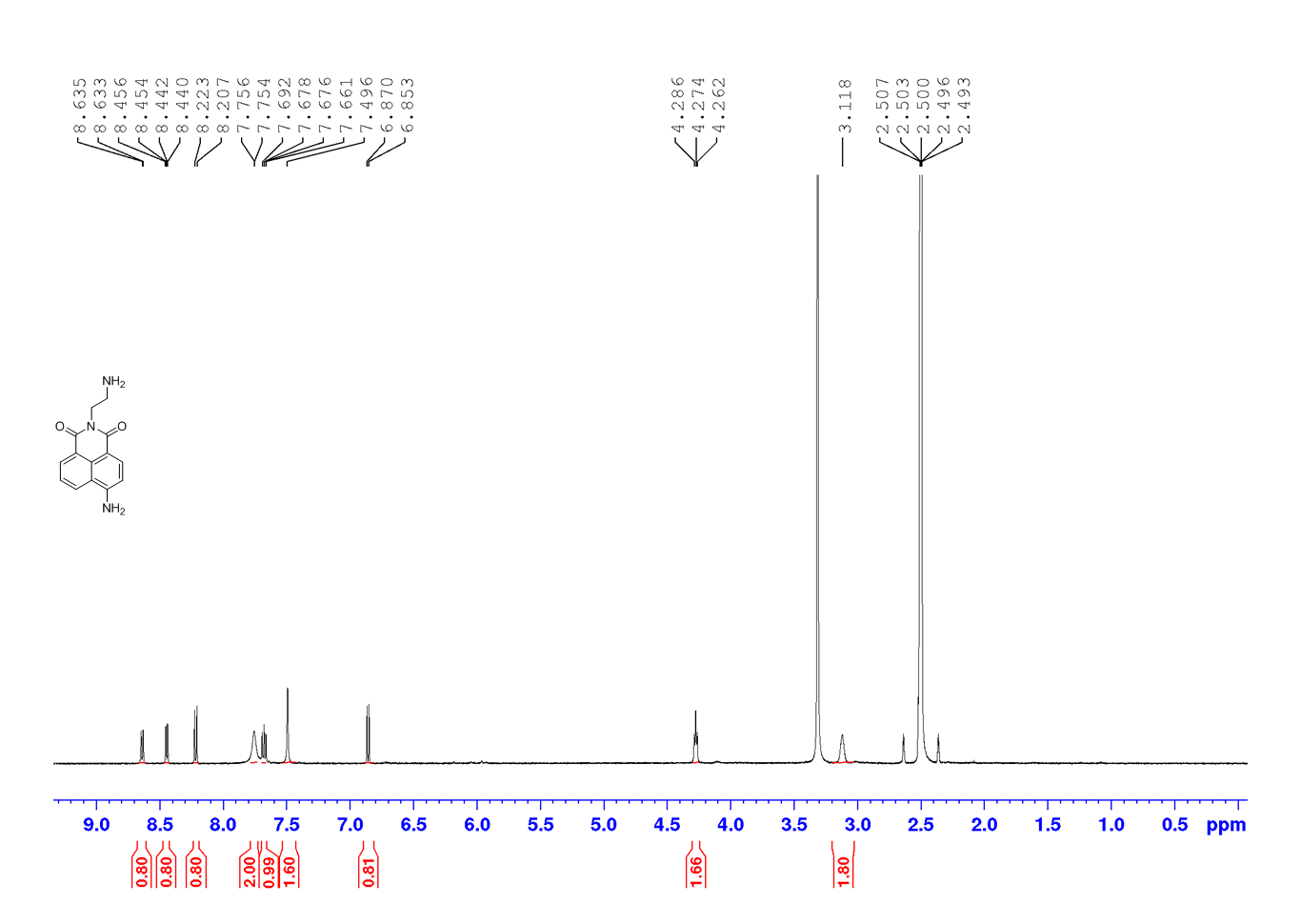


***Figure S15:*** *The ^1^H NMR spectra of* ***3d*** *at 298K (500 MHz, DMSO-_d6_)*


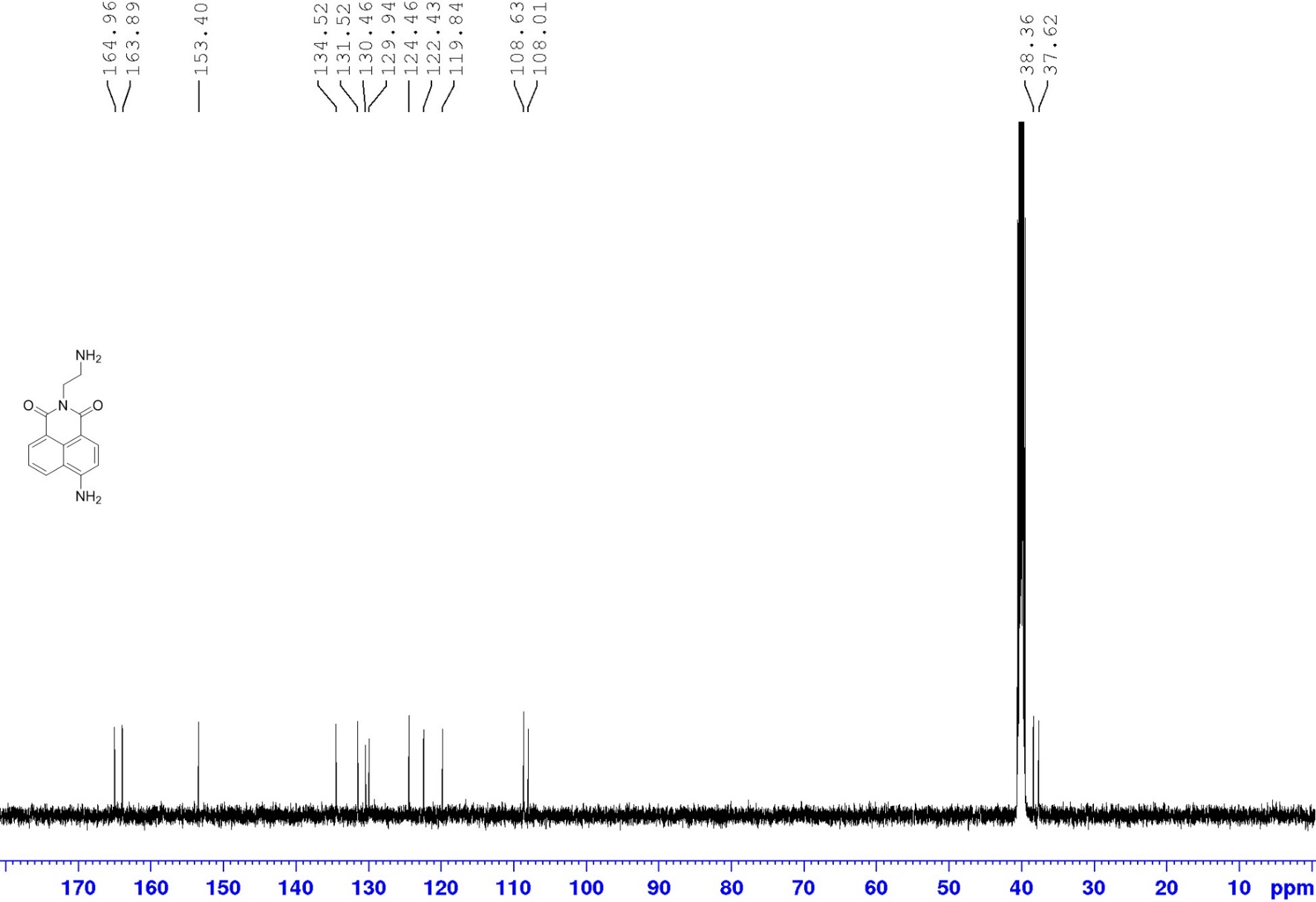


***Figure S16:*** *The ^13^C NMR spectra of* ***3d*** *at 298K (125 MHz, DMSO-_d6_)*


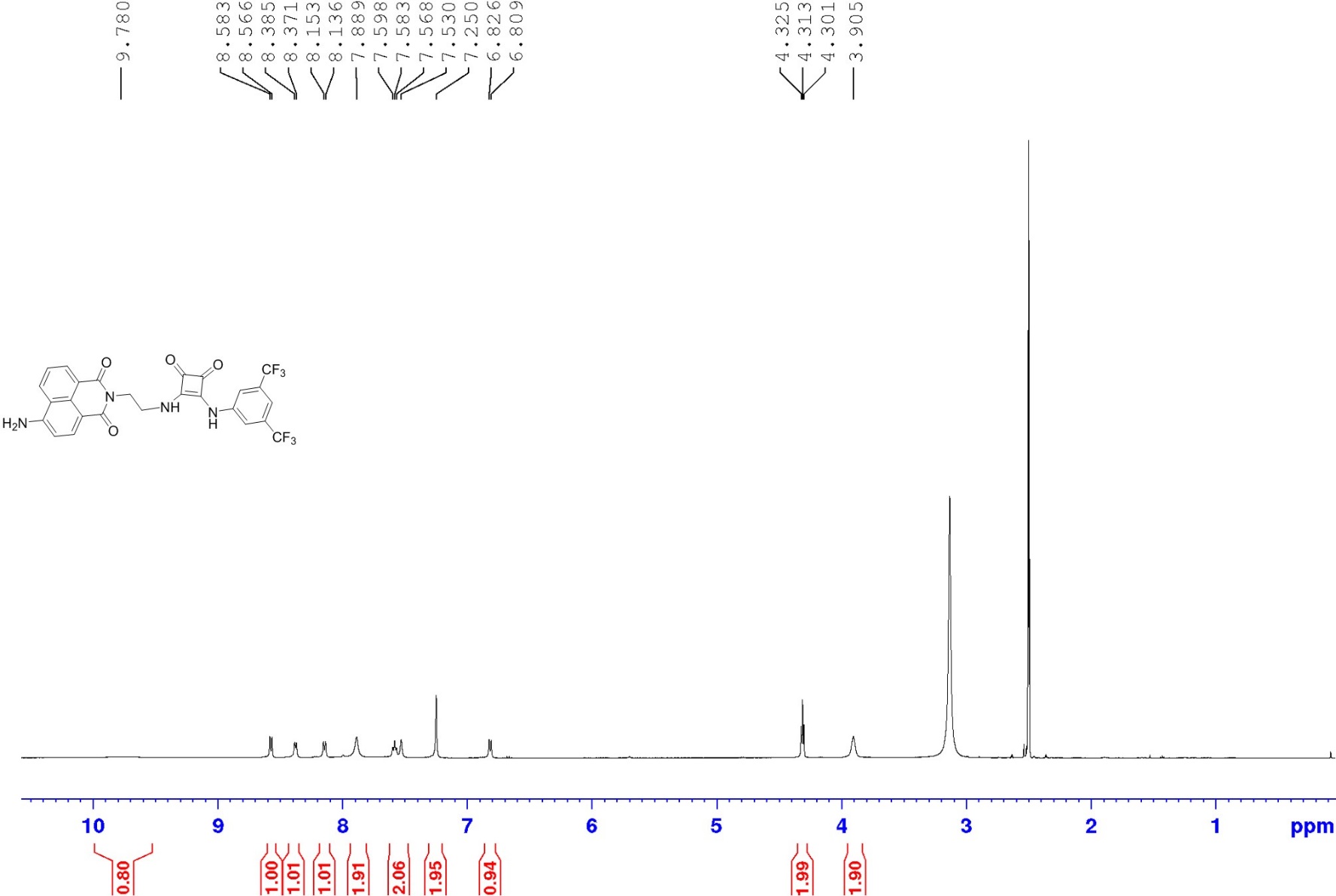


***Figure S17:*** *The ^1^H NMR spectra of* ***SQ2*** *at 343K (500 MHz, DMSO-_d6_)*


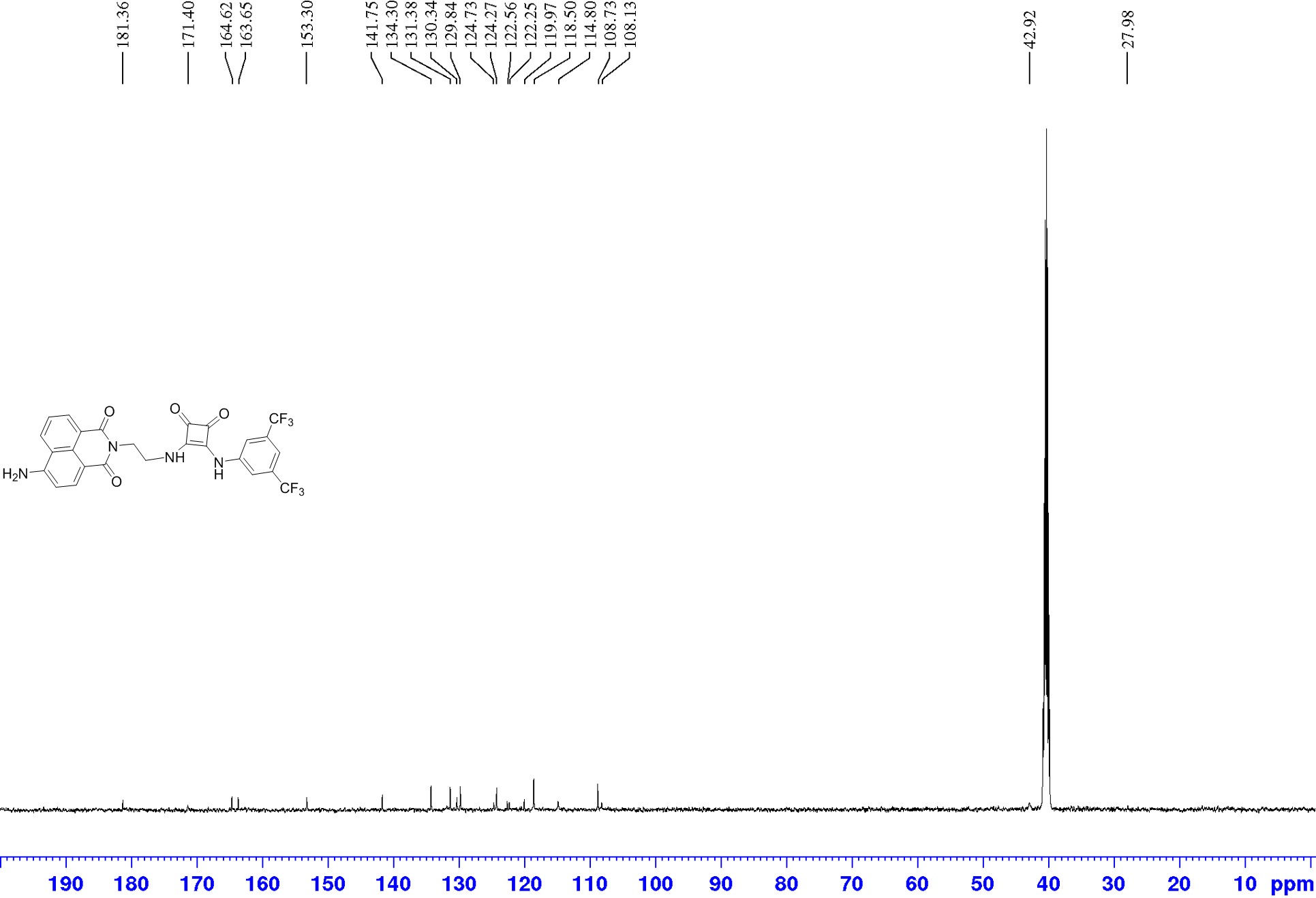


***Figure S18:*** *The ^13^C NMR spectra of* ***SQ2*** *at 343K (125 MHz, DMSO-_d6_)*

***Figure S19:*** *Disaggregation response of probe* ***SQ1*** *(5.0* x *10^–3^ M) at different temperatures. in DMSO-d_6_.*

***Figure S20:*** *Disaggregation response of probe* ***SQ1*** *(5.0* x *10^–3^ M) at different temperatures* *and in the presence of Cl^-^ and Br^-^ in DMSO-d_6_ showing the aliphatic region.*

***Figure S21:*** *Disaggregation response of probe* ***SQ2*** *(5.0* x *10^–3^ M) at different temperatures and in the presence of Cl^-^ and Br in DMSO-d_6_ showing the aliphatic region.*

***Figure S22:*** *Fluorescence temperature study demonstrating the disaggregation response of* ***SQ2*** *(5.0* x *10^–6^ M) in 5% aq. DMSO from 25**° – 110° C.*

***Figure 23:*** *Time dependant fluorescence study showing that the fluorescence intensity of* ***SQ1*** *and* ***SQ2*** *at 525 nm initially increases upon heating to 100 °C but does not decrease upon cooling over the course of 2 hrs.*

***Figure 24:*** *Fluorescence dilution study showing that the fluorescence intensity of* ***SQ1*** *(****●****) and* ***SQ2*** *(****●****) at 525 nm is linear to concentration (from 0.05 µM to 5 µM).*

***Figure S25:*** *Changes in the ^1^H NMR spectrum of* ***SQ1*** *and* ***SQ2*** *(2 mM) upon the addition of 30 equiv. of anions as TBA salts in DMSO-_d6_ ( AcO^–^, Cl^–^, F^–^, H_2_PO_4_^–^, Br^–^, I^–^ and SO_4_^2-^).*

(a)

(b)

***Figure 26:*** *Fluorescence response of (a)* ***SQ-1*** *and (b)* ***SQ-2*** *(5 µM) in 5% aq. DMSO toward different TBA anions (20 mM). (**λ_ex_. 435 nm, λ_em_. 525 nm).*

(a)

(b)

***Figure 27:*** *Fluorescence response of (a)* ***SQ-1*** *and (b)* ***SQ-2*** *(5 µM) in dry DMSO toward different TBA halides (20 mM). (λ_ex_. 435 nm, λ_em_. 525 nm).*

***Figure 28:*** *Fluorescence response of (a)* ***SQ-1*** *and (b)* ***SQ-2*** *(5 µM) in 20% aq. DMSO toward different TBA halides (20 mM). (λ_ex_. 435 nm, λ_em_. 525 nm).*

(a)

(b)

***Figure 29:*** *Fluorescence titration of* ***SQ1*** *(5μM) with different concentration of (a) F^–^ and (b) Cl^–^ in 5% aq. DMSO. λ_exc_ = 435 nm.*

(a)

(b)

***Figure 30:*** *Fluorescence titration of* ***SQ1*** *(5μM) with different concentration of anions: (a) Br^–^ and (b) I^–^  in 5% aq. DMSO. λ_exc_ = 435 nm.*

(a)

(b)

***Figure 31:*** *Fluorescence titration of* ***SQ2*** *(5μM) with different concentration of (a) F^–^ and (b) Cl^–^ in 5% aq. λ_exc_ = 435 nm.*

(a)

(b)

***Figure 32:*** *Fluorescence titration of* ***SQ2*** *(5μM) with different concentration of anions: (a) Br^–^ and (b) I^–^  in 5% aq. DMSO. λ_exc_ = 435 nm.*

(a)

(b)

***Figure 33:*** *Changes in fluorescence response of (a)* ***SQ1*** *and* *(b)* ***SQ2*** *probes in the presence of different halogen anions of TBA salt in 5% aq. DMSO at 525 nm.*


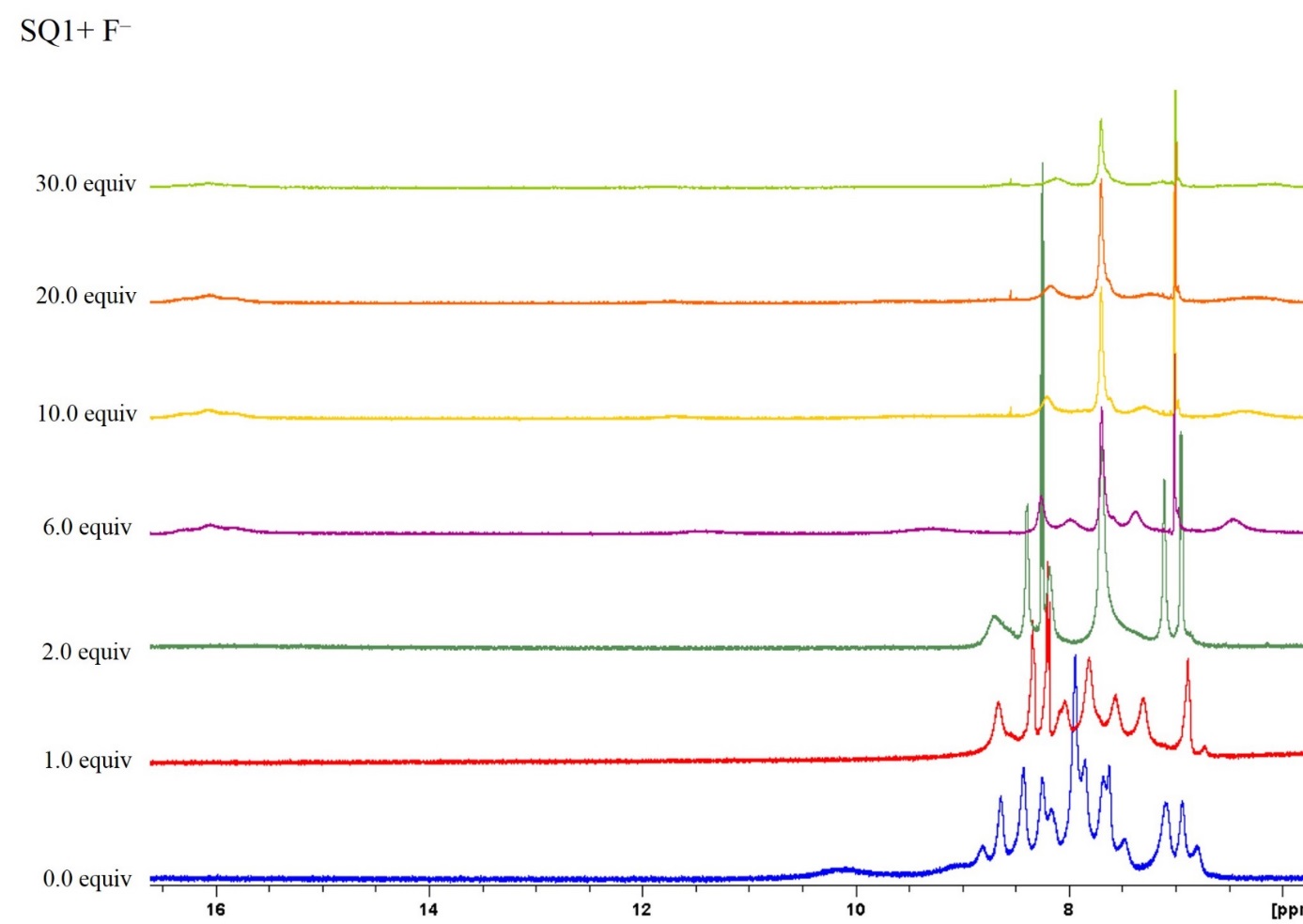


***Figure 34:*** *Changes in the NMR spectra of* ***SQ1*** *(2 × 10^–3^ M) with increasing TBA fluoride concentration in d_6_-DMSO.*


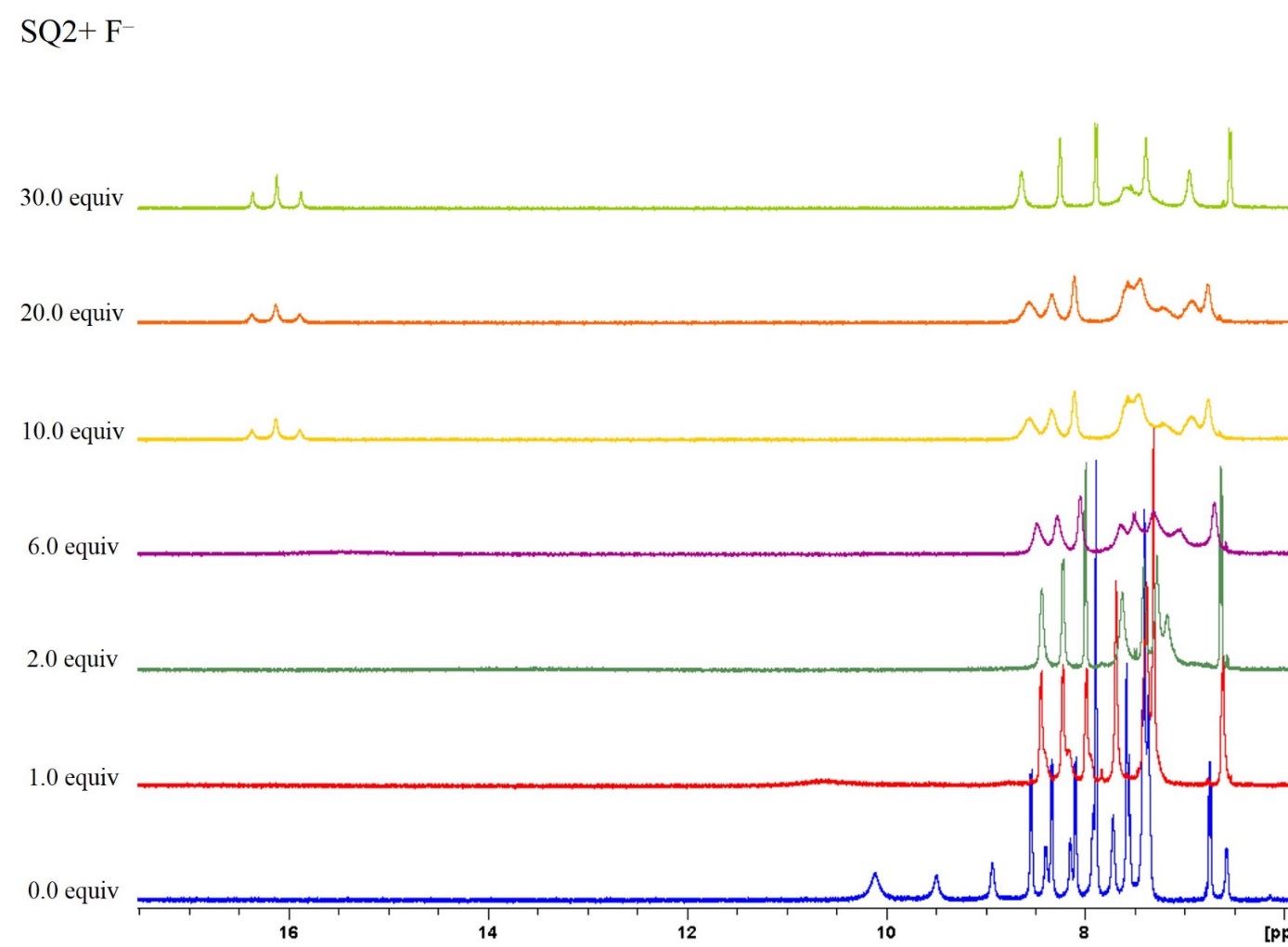


***Figure 35:*** *Changes in the NMR spectra of* ***SQ2*** *(2 × 10^–3^ M) with increasing TBA fluoride concentration in d_6_-DMSO.*


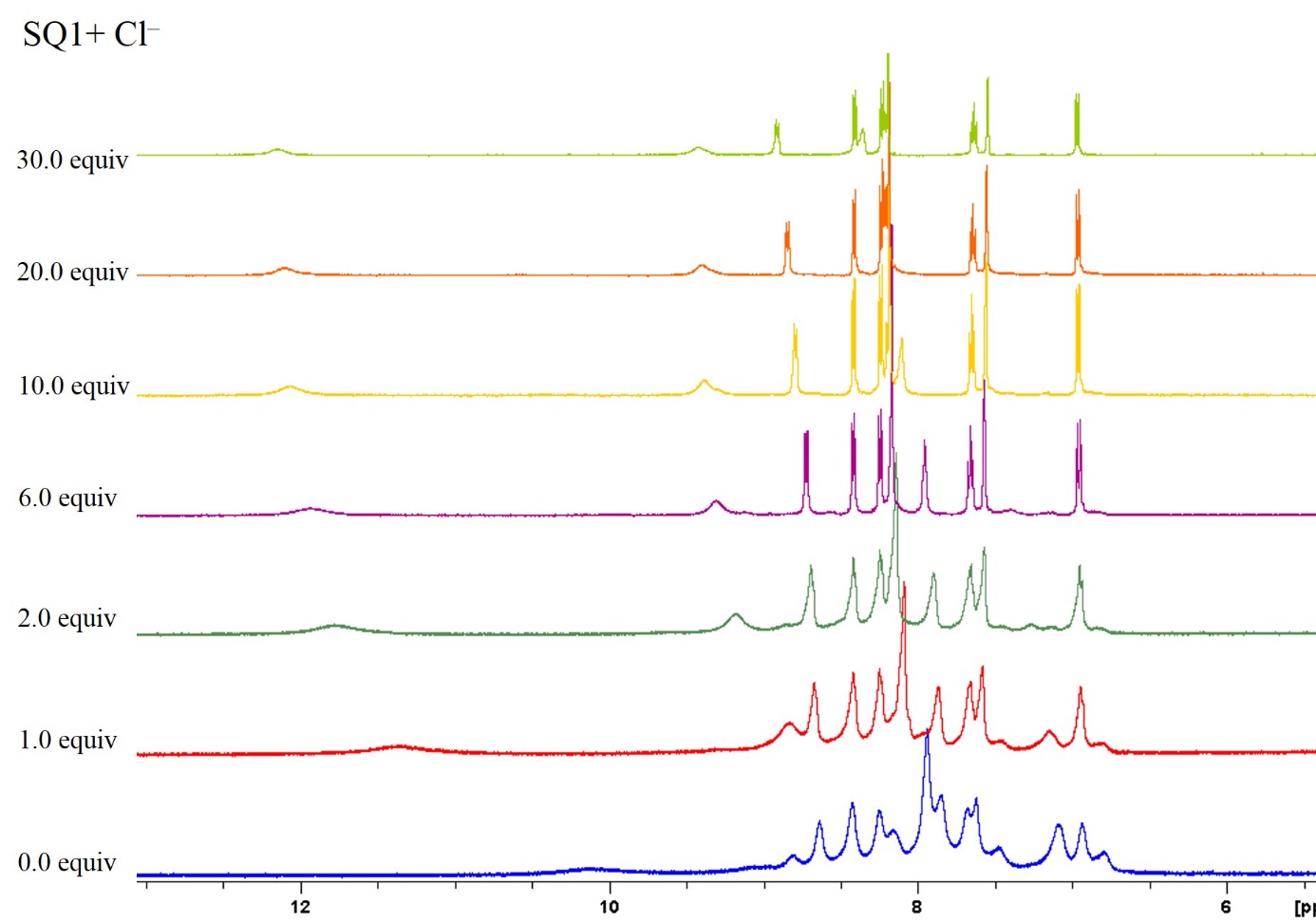


***Figure 36:*** *Changes in the NMR spectra of* ***SQ1*** *(2 × 10^–3^ M) with increasing TBA chloride concentration in d_6_-DMSO.*

*
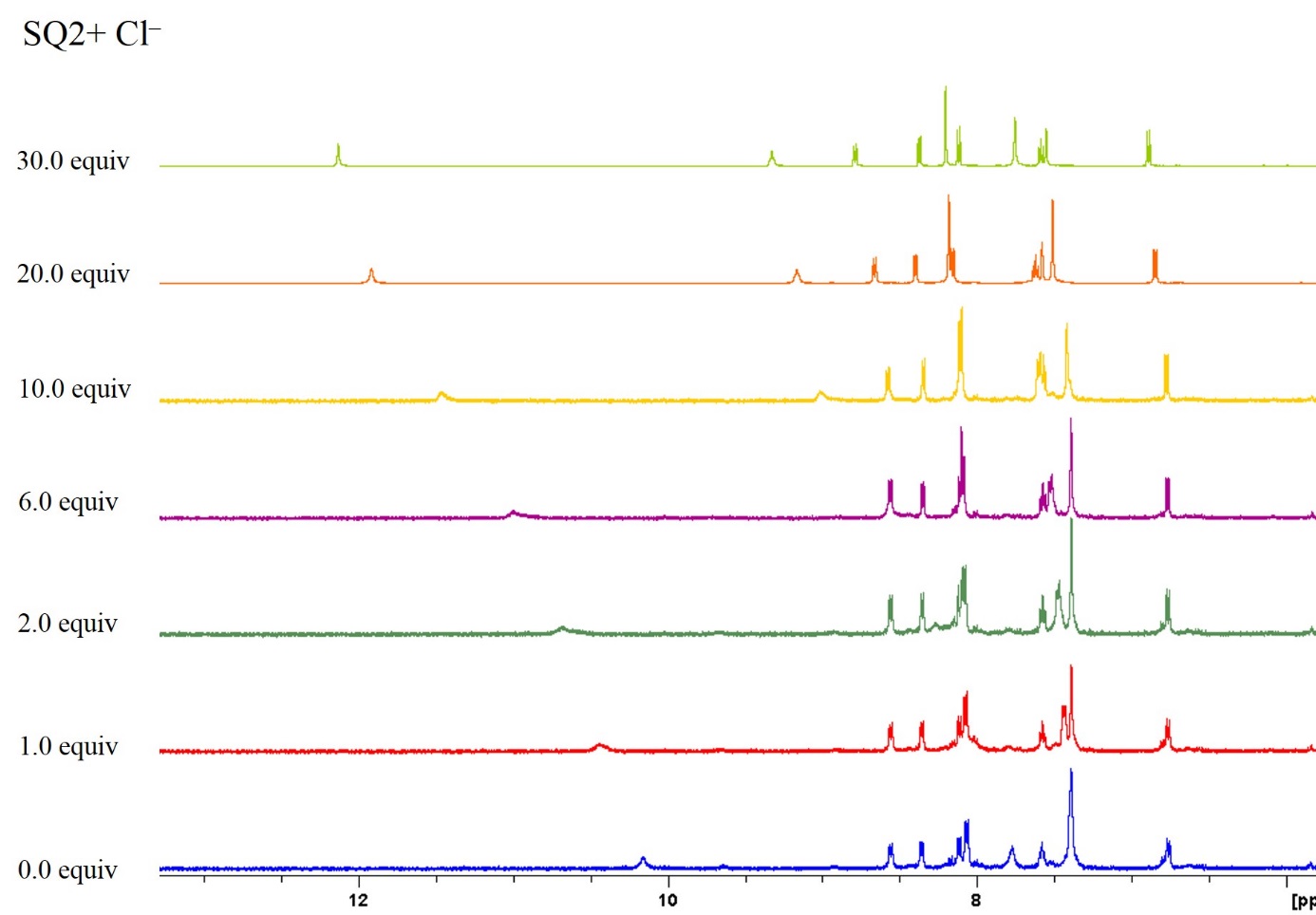
*

***Figure 37:*** *Changes in the NMR spectra of* ***SQ2*** *(2 × 10^–3^ M) with increasing TBA chloride concentration in d_6_-DMSO.*


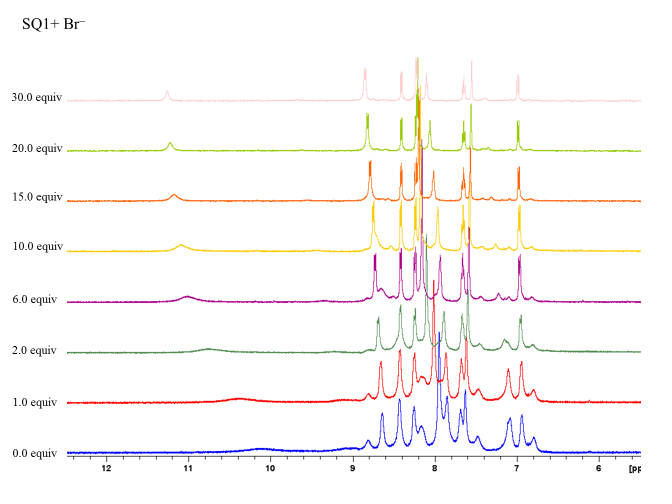


***Figure 38:*** *Changes in the NMR spectra of* ***SQ1*** *(2 × 10^–3^ M) with increasing TBA bromide concentration in d_6_-DMSO.*


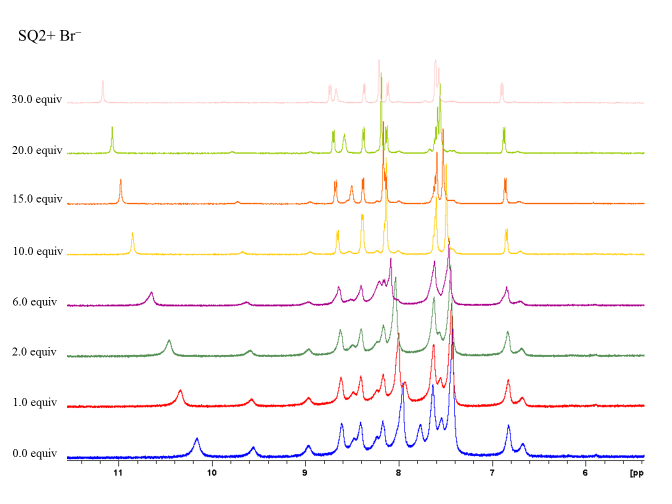


***Figure 39:*** *Changes in the NMR spectra of* ***SQ2*** *(2 × 10^–3^ M) with increasing TBA bromide concentration in d_6_-DMSO.*


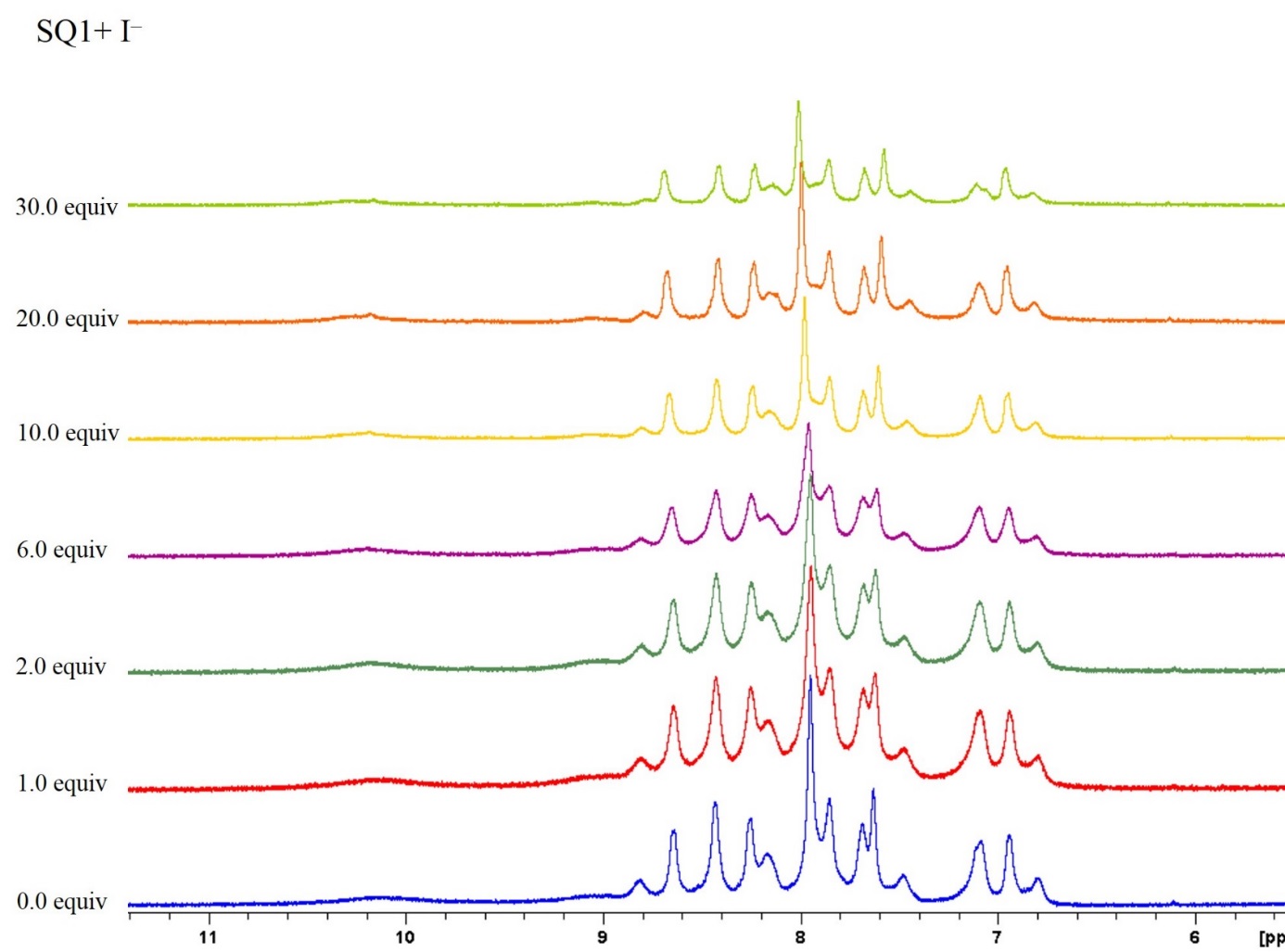


***Figure 40:*** *Changes in the NMR spectra of* ***SQ1*** *(2 × 10^–3^ M) with increasing TBA iodide concentration in d_6_-DMSO.*


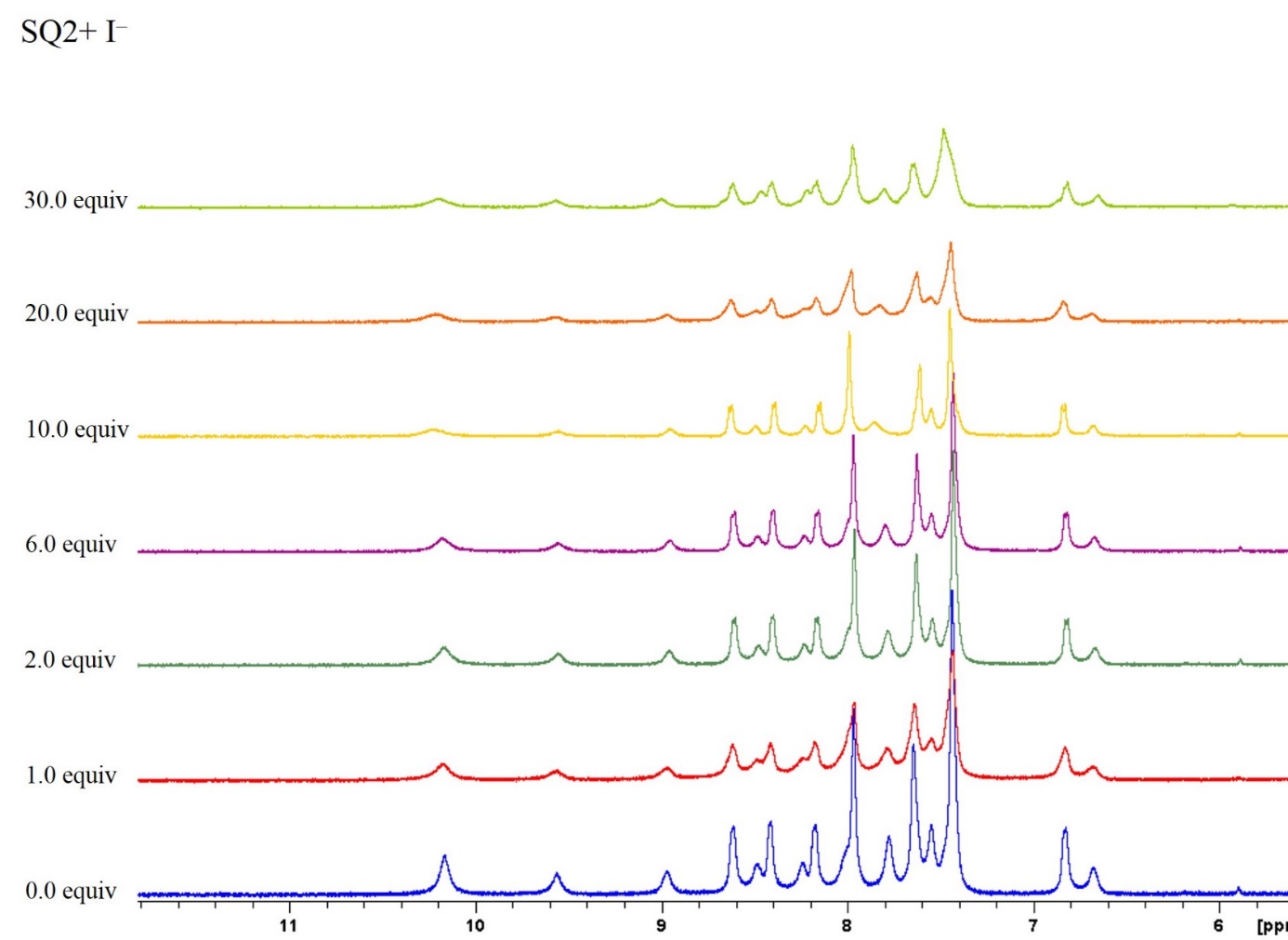


***Figure 41:*** *Changes in the NMR spectra of* ***SQ2*** *(2 × 10^–3^ M) with increasing TBA iodide concentration in d_6_-DMSO.*


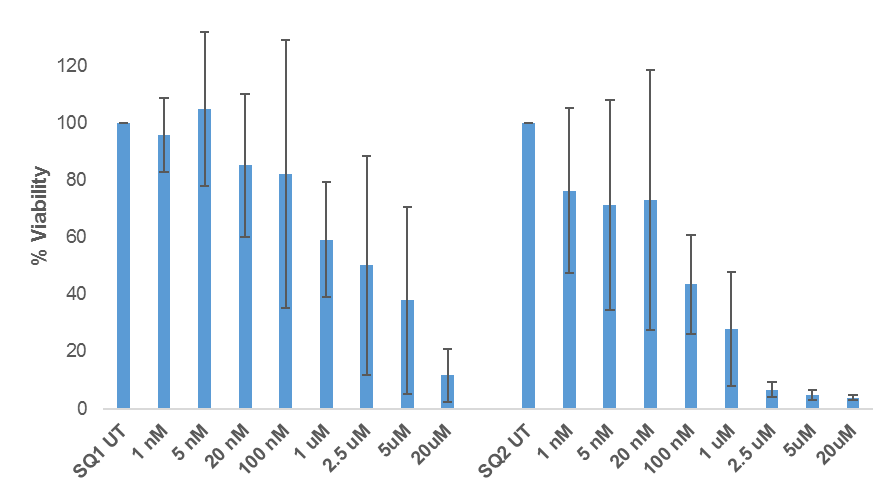


***Figure 42:*** *% Viability data for untreated HeLa cells vs. HeLa cells incubated with* ***SQ1*** *and* ***SQ2*** *over a range of concentrations* *(1 nm – 20 µM) demonstrating the dose-dependent cytotoxicity. Shown is mean data from three independent repeat experiments (+/- stdev).*


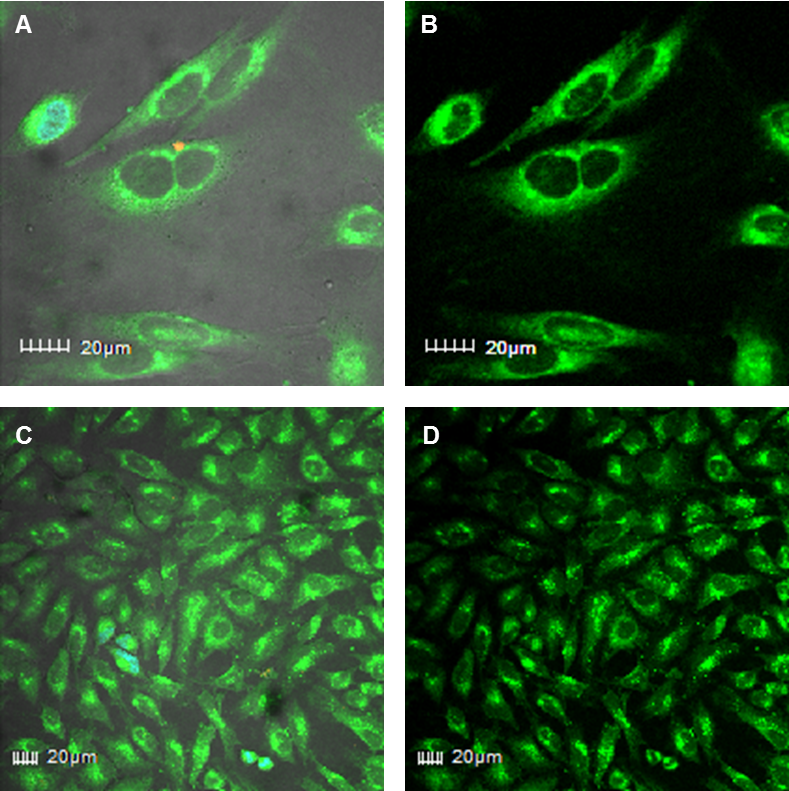


***Figure 43:*** *Uptake of* ***SQ1*** *(20 µM) by HeLa cell. (A – D) shows images obtained by confocal microscopy. (A and C) shows an overlay of the bright field image of treated cells with* ***SQ1*** *(green), (B and C) shows* ***SQ1*** *fluorescence alone (green), Scale bars: 20 μm.*

***
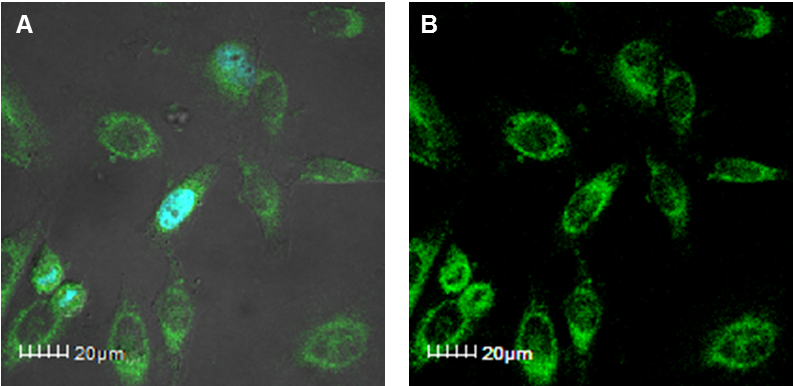
***

***Figure 44:*** *Uptake of* ***SQ2*** *(5 µM) by HeLa cells. (A and B) shows images obtained by confocal microscopy. (A) shows an overlay of the bright field image of treated cells with the DAPI signal (blue) and* ***SQ2*** *(green), (C) shows* ***SQ2*** *fluorescence alone (green), Scale bars: 20 μm.*

**References:**

(1) Liu, H.; Tomooka, C. S.; Moore, H. W. An Efficient General Synthesis of Squarate Esters. *Synth. Commun.* **1997**, *27*, 2177-2180.

(2) Brynn Hibbert, D.; Thordarson, P. The death of the Job plot, transparency, open science and online tools, uncertainty estimation methods and other developments in supramolecular chemistry data analysis. *Chem. Commun.* **2016**.

(3) Thordarson, P. Determining association constants from titration experiments in supramolecular chemistry. *Chem. Soc. Rev.* **2011**, *40*, 1305-1323.

(4) Lowe, A. J.; Pfeffer, F. M.; Thordarson, P. Determining binding constants from 1H NMR titration data using global and local methods: a case study using [n]polynorbornane-based anion hosts. *Supramol. Chem.* **2012**, *24*, 585-594.
